# Oxidative Stress after Pollutant Exposure Depends Strongly on Experimental Design and Pollutant Properties in Annelids: A Meta-Analysis

**DOI:** 10.1021/acs.est.6c03847

**Published:** 2026-08-13

**Authors:** Max V. R. Döring, Heike Feldhaar, Ana L. Antonio Vital, Magdalena M. Mair

**Affiliations:** † Animal Population Ecology, Animal Ecology I, 26523 University of Bayreuth, 95440 Bayreuth, Germany; ‡ Bayreuth Center of Ecology and Environmental Research (BayCEER), 95440 Bayreuth, Germany; § Statistical Ecotoxicology, University of Bayreuth, 95440 Bayreuth, Germany

**Keywords:** oxidative distress, contamination, ecotoxicology, annelids, physico-chemical properties, statistical power

## Abstract

Measurements of oxidative stress are often performed to assess a species’ general sublethal stress response to a pollutant. However, oxidative stress bioassays often produce seemingly ambiguous results, and the drivers that lead to these differences are largely unknown. To address this gap, we conducted a meta-analysis on reactive oxygen species (ROS) generation, ROS-associated damage products, enzyme activities, and gene expression levels in response to exposures to two groups of pollutants, nano- and microplastic particles (NMP) and neonicotinoid insecticides (neonics). Based on 2294 ROS-related measurements extracted from 45 studies, we show that measured effects vary substantially, with a strong overlap of measured effects with zero. As likely drivers of this variance, we identified multiple parameters of experimental design and pollutant properties. Finally, we performed data simulations and power analyses to investigate how well single experiments are able to detect oxidative stress-related effects. While most oxidative stress markers achieve sufficient power (80%) to demonstrate effects with sample sizes *N* < 20, conclusions derived from single studies with low sample sizes (*N* < 5) are at risk of being less informative than previously assumed. Our results highlight the importance of considering pollutant properties, experimental design, and sample size when measuring oxidative stress markers.

## Graphical abstract

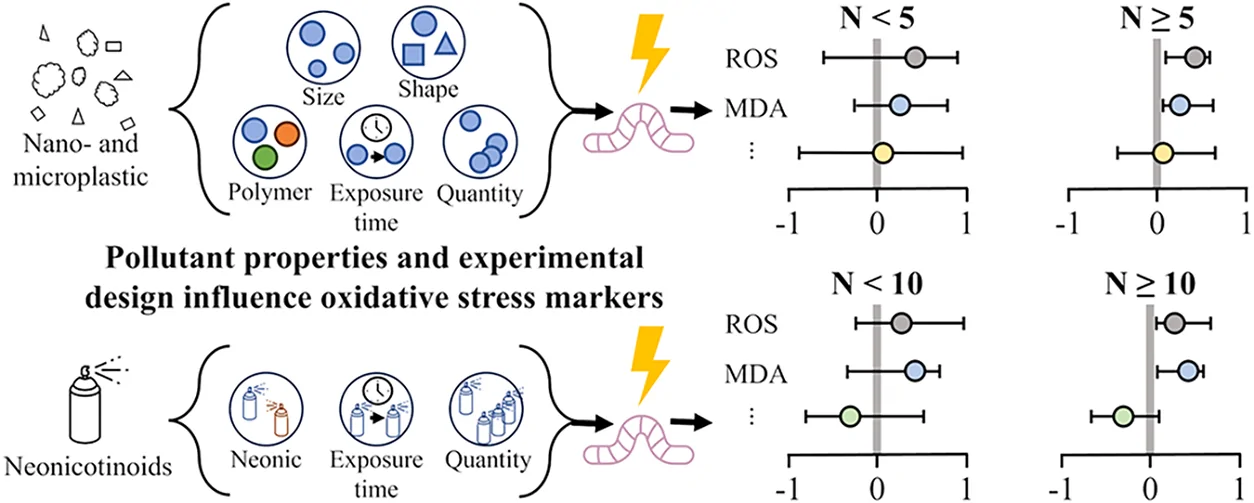

## 1 Introduction

Reactive oxygen species (ROS) are a class of molecules derived from oxygen (O_2_) that are more reactive than O_2_ itself.^1^ ROS are naturally produced within cells by metabolic processes, including the mitochondrial respiratory chain^2,3^ and NADPH oxidases,^4^ and are important as redox signaling agents.^5,6^ ROS such as hydrogen peroxide have the potential to cause growth arrest and cell death in high concentrations,^5^ so their levels inside organisms are controlled tightly. This is achieved by regulating the rate of endogenous ROS generation,^7^ by increasing ROS scavenging via upregulation of antioxidant production (e.g., glutathione)^8^ and ROS scavenging enzyme expression and activity,^9^ or via uptake of nonenzymatic antioxidants, e.g., vitamin C and E.^10^ However, intracellular ROS levels can additionally be increased by external stressors, overwhelming the ROS scavenging capabilities and leading to oxidative stress and subsequent damage.^11,12^ Consequently, to assess the impact of various external stressors such as pollutants on organisms, it is common practice to monitor various oxidative stress markers, including indicators of pro-oxidant status, antioxidant defenses, and cellular damage products. Increased levels of these markers are interpreted as increased oxidative stress and, consequently, higher toxicity of the tested external stressor.

When reading ecotoxicological literature, we observed that the same oxidative stress markers are often reported to be affected in different directions in different studies (e.g., one study showing an increased enzyme activity while another study shows a decreased activity of the same enzyme; see examples in Table 1). This observed variability raises questions about its underlying drivers. For instance, specific oxidative stress markers might be up- or downregulated depending on factors such as pollutant type, pollutant properties, and experimental conditions (e.g., exposure time). Given the high variance in observed effects, it is additionally unclear whether currently used experimental designs have sufficient statistical power (i.e., the rate at which true effects can indeed be detected^13^) to reliably detect true effects.

**Table 1:**
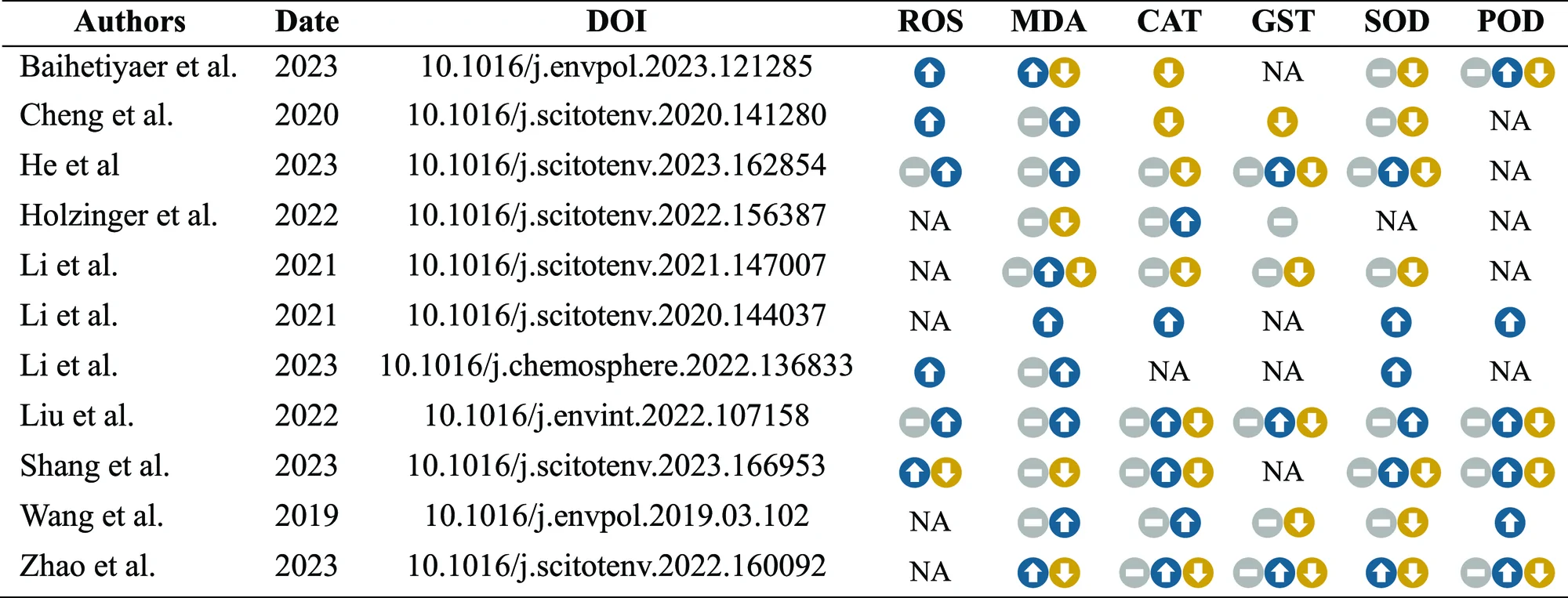
Reactive Oxygen Species (ROS) Concentrations, Damage Products, and Antioxidant Enzymes are Highly Variable within and among Studies^a^

a 11 example studies from our data set that measured ROS concentration, malondialdehyde (MDA) concentration, catalase (CAT) activity, glutathione-S-transferase (GST) activity, superoxide dismutase (SOD) activity, and/or peroxidase (POD) activity in annelids after nano- and microplastic (NMP) exposure. As examples, we chose studies from our data set that measured at least three of the six oxidative stress markers. Upwards pointing arrows with a blue background symbolize reported significant increases in enzyme activity or molecule concentration compared to the control, downwards pointing arrows with a yellow background symbolize a significant decrease. Hyphens with a grey background represent no significant difference between NMP treatment and control. Enzyme activities or concentrations that were not determined are shown as NA. Most enzyme activities or molecule concentrations show mixed results within the same oxidative stress marker and study when multiple exposure times, concentrations, shapes, sizes, or plastic ages were tested.

The aim of our study was therefore to uncover general patterns of oxidative stress marker responses when organisms are confronted with different pollutants by using a meta-analytic approach. Such patterns will then, on the one hand, allow a more efficient choice of oxidative stress markers in future studies, as the most responsive ones can be characterized. On the other hand, general and recurring patterns form the basis for future investigations and interpretations of the mechanisms underlying the respective responses. By comparing patterns of oxidative stress markers of two different pollutant groups, nano- and microplastic particles (NMPs) and neonicotinoid insecticides (neonics), we aim to test whether oxidative stress markers respond in pollutant-specific patterns, which may arise due to the different modes of action of these two pollutant groups.

NMPs are omnipresent particulate pollutants that have received increasing attention in the last 20 years, while their number in the environment is steadily increasing.^14^ They elicit mostly sublethal effects, but the mechanisms of how NMP leads to oxidative stress are not sufficiently well understood and likely include both particle-induced effects (e.g., physical damage and subsequent activation of the immune system^15^) and chemical-induced effects (e.g., through plastic-associated chemicals^16^). Neonics have been one of the most used classes of agricultural insecticides worldwide^17^ with well-documented negative effects on various nontarget organisms.^18^ They were designed to open excitatory nicotinic acetylcholine receptors and cause paralysis due to overstimulation of neurons.^19^ However, they also seem to induce increased ROS levels in target and nontarget organisms by multiple pathways^20^ such as disruption of Ca^2+^ homeostasis^21,22^ and potentially by altering key ROS regulatory genes.^23^ The extent to which different neonics activate these pathways remains unclear.

In total, we extracted 2294 ROS-related measurements from 45 annelid studies (see Section 2.1). Annelid worms were chosen since they are established model organisms in ecotoxicology and are very sensitive to changes in their environment.^24^ We examined (1) the directions and strengths of average effects of NMP and neonics on different oxidative stress markers and (2) the explanatory power of pollutant properties and experimental design choices on the observed variance. Based on the estimated average effect sizes and variances, we (3) performed extensive data simulations and power analyses to estimate the statistical power of typical test designs and the sample sizes required to detect true effects at sufficiently high rates (i.e., with a statistical power of 0.8). To this end, we first assumed a general oxidative stress-related response and, second, inspired by the empirical results, allowed for differences in the true stress response due to differences in experimental design parameters (e.g., concentration, exposure duration, and sample size) and pollutant properties (e.g., polymer type for NMP or type of neonic).

We extracted oxidative stress-related measurements (e.g., enzyme activity measurements), experimental design parameters (e.g., number of replicates), and reported statistical outcomes from 21 and 24 studies examining the effects of NMP and neonics on annelids, respectively. The log-transformed ratio of means (logROM) for differences between treatments (numerator) and controls (denominator) was calculated as the effect size. Overall average effects of different pollutants on measured oxidative stress markers (i.e., one effect size estimate per oxidative stress marker for NMP and neonics separately) were derived from mixed meta regression models^25^ (see Section 2.2). The following oxidative stress markers were evaluated: ROS formation (overall ROS and hydroxyl radical (^•^OH) concentration), oxidative stress responses (enzyme activities of catalase (CAT), superoxide dismutase (SOD), glutathione-S-transferase (GST), peroxidase (POD), glutathione reductase (GR), carboxylesterase (CarE), mRNA expression levels of CAT, SOD, and GST, total antioxidant capacity (TAC), glutathione (GSH) concentration), and oxidative stress-associated damage products (malondialdehyde (MDA), 8-hydroxy-2-deoxyguanosine (8-OHdG), and protein carbonyl group (PC) concentrations).

## 2 Methods

### 2.1 Data Collection

We conducted a systematic search for all peer-reviewed publications that were published before December 2023, and that examined the effects of NMP or neonics on ROS generation, antioxidant enzyme activities, and oxidative stress-associated damage products in terrestrial annelids. We started the search for suitable articles by choosing 10 already published, representative articles for each NMP and neonics. Afterward, we optimized our search string such that these 10 previously selected articles appeared in the 50 most relevant (i.e., the first 50) papers on Web of Science (https://www.webofscience.com). For search term optimization, different combinations of keywords were tested, including terms related to oxidative stress responses (e.g., ROS, oxidative stress, reactive oxygen species, detoxification, and names of specific oxidative stress markers), terms associated with the focal taxon of annelid worms (e.g., annelid, Eisenia, earthworm), and terms describing NMP (e.g., microplastic*, nanoplastic*) and neonicotinoids as focal pollutant types (e.g., neonic* and different specific neonicotinoid names). Finally, on the 14th and 15th of December 2023, we searched on Web of Science with the following search string for NMP (49 hits): “(annelid* OR eisenia) AND (reactive oxygen species OR oxidative stress) AND (microplastic* OR nanoplastic*)” and neonics-associated studies (51 hits), respectively: “(annelid* OR eisenia OR earthworm*) AND (oxidative stress OR detoxification OR inhibition) AND (neonic* OR imidaclo* OR thiaclo*)”.

The titles and abstracts of all publications analyzing the effects of NMP or neonics were screened to fulfill the following criteria: The studies investigated the oxidative stress of terrestrial annelids following an *in vivo* pollutant exposure (i.e., no single-cell analyses) and included a negative control without the respective pollutant. We excluded reviews and meta-analyses. Studies using tire wear particles (TWPs) or in which the worms were coexposed to other pollutants in addition to NMP or neonics were excluded to avoid confounding by additional substances (e.g., softener and vulcanization agents in TWPs^26^). We also excluded studies that did not report essential data for statistical analysis (e.g., missing number of biological replicates) and where the authors had not responded to a request for raw data. Lastly, for improved comparability within our NMP data, we excluded data generated after a recovery period (i.e., period after the pollutant exposure without pollutant; 84 data points) and data obtained through a neonic contact test (i.e., annelids are exposed to the neonic on a filter paper in Petri dishes) from our main analysis (174 data points). For the latter, we included a separate analysis in the Supporting Information (Table S1 and Figure S1). This left us with 21 NMP studies (898 data points) and 24 neonic studies (1396 data points). For a visual representation (PRISMA (Preferred Reporting Items for Systematic Reviews and Meta-Analyses) flow diagram) of the article selection process, see Figure S2.

From each of the remaining articles, we extracted data regarding the species and life stage of annelids at the start of exposure, the exposure route (whether the pollutants were mixed in the soil or food or if a contact test was performed), type of soil (artificial or collected from the environment), soil parameters (pH and mean temperature), exposure and recovery duration, nominal and measured pollutant concentration, added chemicals, screening for chemical and NMP contamination of the soil, uptake validation (whether pollutant uptake was verified) and validation method, food type and dose, measured oxidative stress marker and respective unit, measurement method, the number of biological and technical replicates, and the pollutant manufacturer. The extracted oxidative stress markers were measured ROS levels, antioxidant enzyme activities (CAT, SOD, GST, POD, GR, CarE), and some of their mRNA expression levels, GSH concentrations, the TAC, and concentrations of oxidative stress-induced cell damage products (MDA, Protein carbonyl, 8-OHdG). For NMP, we additionally extracted whether NMP were cleaned prior to exposure and the solvent used for cleaning, the polymer type, shape, nominal and measured mean particle size, and, if applied, the method of aging (including artificial UV-weathering and exposure to the environment prior to their use in exposure bioassays). For neonics, we determined the specific neonicotinoid identity (i.e., the name of the chemical) that was used. Oxidative stress marker measurements were preferentially extracted from the text or calculated from raw data. If this was not possible, measurements were extracted from figures using the *metaDigitise* package (version 1.0.1)^27^ in R (version 4.3.1).^28^ All extracted data were double-checked and validated by a second person, and discrepancies were discussed until a consensus was reached.

### 2.2 Data Analysis

Units of all measured concentrations, enzyme activities, and expression levels were converted into nine defined units (fluorescence intensity, fluorescence intensity/mg protein, nmol/mg protein, nmol/mL homogenate, U/mg protein, ng/g fresh weight, ng/l, ng/mg DNA, and fold change). In case results were reported as a fold change and data were obtained via the *metaDigitise* package, we set the mean of the control per definition to 1. We did not do this for 48 data points of one study, where the measurements were reported as fold change, but the mean value of the control was clearly different from 1. Two articles^29,30^ quantified the thiobarbituric acid reactive substances concentration as a proxy for lipid peroxidation. These oxidative stress markers were treated as malondialdehyde concentrations.^31^ All pollutant concentrations were log-transformed and converted to weight percentages (% w/w). If the nominal as well as the measured concentration or size were reported, we used the measured concentration in our analysis. The NMP concentration in one article (2 data points) could not be converted, as it was only reported in particles per kg of soil. These two data points were neglected in all models considering the NMP concentration. Most articles that measured enzyme activity and expression levels did not report the exact enzymes or genes they investigated. Therefore, we summarized the genes by their function (CAT, SOD, GST, POD, CarE, and GR) rather than their specific names (e.g., glutathione peroxidase 1).

All statistical analyses were done in R (version 4.3.1).^28^ The data were filtered using the package *dplyr* (version 1.1.4).^32^ For each treatment-control pair, we calculated the log-transformed ratio of means (logROM) as an effect size and the corresponding sampling variance (vi) using the *metafor* package (version 4.8–0).^25^ We excluded data points where both the control and treated worms had measured values of zero (9 cases in total), which made it impossible to calculate the logROM. We also excluded data points with a sampling variance of zero (20 cases in total) since the model cannot calculate the proper weight of these data points, and a true variance of zero seems highly unlikely. To assess whether the exclusion of these data points influenced our results, we repeated all analyses on the complete data set and replaced the zero variances with 10^–7^. This did not change our general results (i.e., no changes in significance; see Figure S3 and Table S2).

We multiplied the logROM by minus one, so that a positive logROM indicates higher measurements of the treatment group compared to the control. Mixed meta regression models without an intercept were fitted to the data to evaluate the effects of NMP and neonics. We included the oxidative stress markers as a fixed effect and a nested random intercept (∼1|DOI/individual_level), where the article’s digital object identifier (DOI) accounted for the heterogeneity between studies, and the individual level random effect accounted for the heterogeneity between samples within studies. The individual level refers to the individual data point level and was generated by assigning each data point with a unique number (individual_level = 1:nrow­(data)) in R. We additionally included a quadratic term and intercept in models that examined the effects of neonics and NMP over time, with different sizes, or at different concentrations. These models were plotted with *metafor* and rated as significant if at least one of the terms had a *P* value below 0.05. Data and models were illustrated using the packages *orchaRd* (version 2.0)^33^ and *ggplot2* (version 4.0.0).^34^ Since the GST mRNA expression was only measured at two time points after NMP exposure (14 and 28 days), we only assumed a linear relationship in these cases and excluded the quadratic terms. Additionally, the effects of different NMP sizes on two oxidative stress markers (TAC and GST mRNA expression) could not be evaluated because only one NMP size was used.

Since some pollutant properties determined the effects they had on certain oxidative stress markers, we wanted to figure out if including the properties as fixed factors in a model would significantly improve its fit compared to models without them. Using likelihood ratio tests (LRT), we compared a full model containing all properties (exposure time, concentration, shape, polymer, and size for the NMP data set, and exposure time, concentration, and neonic identity for the neonic data set) with models where one property at a time was excluded (i.e., reduced models). Additionally, we wanted to figure out if the effects of experimental design and pollutant properties were different among oxidative stress markers. We therefore created a full model with all of the above-mentioned properties plus their interactions with the oxidative stress markers and compared it with reduced models via LRT, where each of the interaction terms was excluded one by one. Finally, we calculated the proportion of the variance in the data that was explained by the pollutant properties and experimental design parameters. To this end, we fitted a model containing only the oxidative stress marker as a fixed effect and calculated the sum of both σ^2^ variance components (σ_end point_^2^). We repeated the procedure with a full model containing all of the above-mentioned properties plus their interactions with the oxidative stress marker (σ_predictors_^2^). Finally, we calculated the residual heterogeneity (i.e., variance in the true effects) by subtracting σ_predictors_^2^ from σ_end point_^2^ and dividing the result by σ_end point_^2^. Since the proportion of the sampling variance to the total unaccounted variance was extremely low (<0.6%), we used the residual variance as an approximation for the total unaccounted variance. We observed a large amount of residual heterogeneity, and our data points did not come from a common true effect, so we intentionally did not conduct a publication bias analysis (e.g., funnel plots^35^).

### 2.3 Power Analysis

We conducted multiple power analyses to determine at which rates commonly performed experiments can detect true effects. To this end, we calculated the means and standard deviations (SD) of each control and treatment group for each oxidative stress marker (NMP: 12 oxidative stress markers times two groups = 24 separate values; neonics: 15 oxidative stress markers times two groups = 30 separate values). Means and SD were then used to draw random samples from normal distributions, both for the control and the treatment group, thus simulating experiments in the laboratory. This procedure was repeated 100 times for each oxidative stress marker-pollutant combination. Each simulated control-treatment pair was then analyzed via a *t* test. The statistical power was finally calculated as the proportion of *t* tests with *P* values equal to or below 0.05. The simulation process was repeated with increasing sample sizes (i.e., the number of random samples drawn) ranging from three to 100 (incremented by one). Finally, we determined the lowest sample size at which the statistical power was equal to or higher than 0.8 (which would translate into significant results in 80% of experiments) and the statistical power at sample sizes of three and eight, which were the most frequently used and highest sample sizes in our data set, respectively.

Considering differential stress responses dependent on experimental design parameters and pollutant properties, we performed a second set of power analyses that took these differences into account. To this end, we calculated the means and SD separately for each combination of pollutant properties and experimental design parameters (NMP: 813 combinations, neonic: 1241 combinations). For each combination, we performed a power analysis as described above with a maximum sample size of 50 instead of 100 (47 sample sizes × 100 *t* tests = 4700 *t* tests per combination). The statistical power for different property-experimental design combinations was averaged for each sample size and oxidative stress marker. Finally, the lowest sample size at which the statistical power was equal to or greater than 0.8 was extracted, along with the statistical power at sample sizes of three and eight.

## 3 Results and Discussion

### 3.1 Effects of Pollutants on Oxidative Stress Markers

In summary, we observed that on average, both pollutant groups increased ROS concentrations and all damage products and significantly altered 8 of the 19 oxidative stress markers. We found that on average, exposure to NMP induced a significant increase in the ROS concentration (mean logROM ± SD: 0.173 ± 0.246, *Z* = 3.265, *P* = 0.001), the CAT mRNA expression level (0.354 ± 0.350, *Z* = 4.444, *P* < 0.001), the GSH concentration (0.184 ± 0.591, *Z* = 2.151, *P* = 0.031), the MDA concentration (0.156 ± 0.385, *Z* = 3.427, *P* < 0.001), and the 8-OHdG concentration (0.199 ± 0.105, *Z* = 3.290, *P =* 0.001) (Figure 1 and Table S3). In contrast, the GST mRNA expression level (−0.178 ± 0.165, *Z* = −1.996, *P* = 0.046) decreased on average. Neonic exposure increased the ROS concentration (mean logROM ± SD: 0.183 ± 0.130, *Z* = 4.462, *P* < 0.001), the ^•^OH concentration (0.177 ± 0.157, *Z* = 2.244, *P* = 0.025), CAT activity (0.096 ± 0.348, *Z* = 2.548, *P* = 0.011), SOD activity (0.148 ± 0.353, *Z* = 3.837, *P* < 0.001), SOD mRNA expression (0.399 ± 0.594, *Z* = 5.790, *P* < 0.001), CAT mRNA expression (0.182 ± 0.595, *Z* = 2.648, *P* = 0.008), the MDA concentration (0.207 ± 0.250, *Z* = 5.241, *P* < 0.001), the 8-OHdG concentration (0.247 ± 0.190, *Z* = 3.432, *P* = 0.001), and the PC concentration (0.166 ± 0.079, *Z* = 2.994, *P* = 0.003) (Table S4). Only the CarE activity (−0.241 ± 0.466, *Z* = −3.173, *P* = 0.002) was significantly decreased under exposure to neonics. So on average, both pollutant groups (NMP and neonics) increased ROS levels and damage product concentrations and altered the gene expression levels of many of the analyzed enzymes and enzyme activities, which is consistent with results from previous meta-analyses examining the effects of NMP^36^ and neonics^18^ on soil biota.

**Figure 1:**
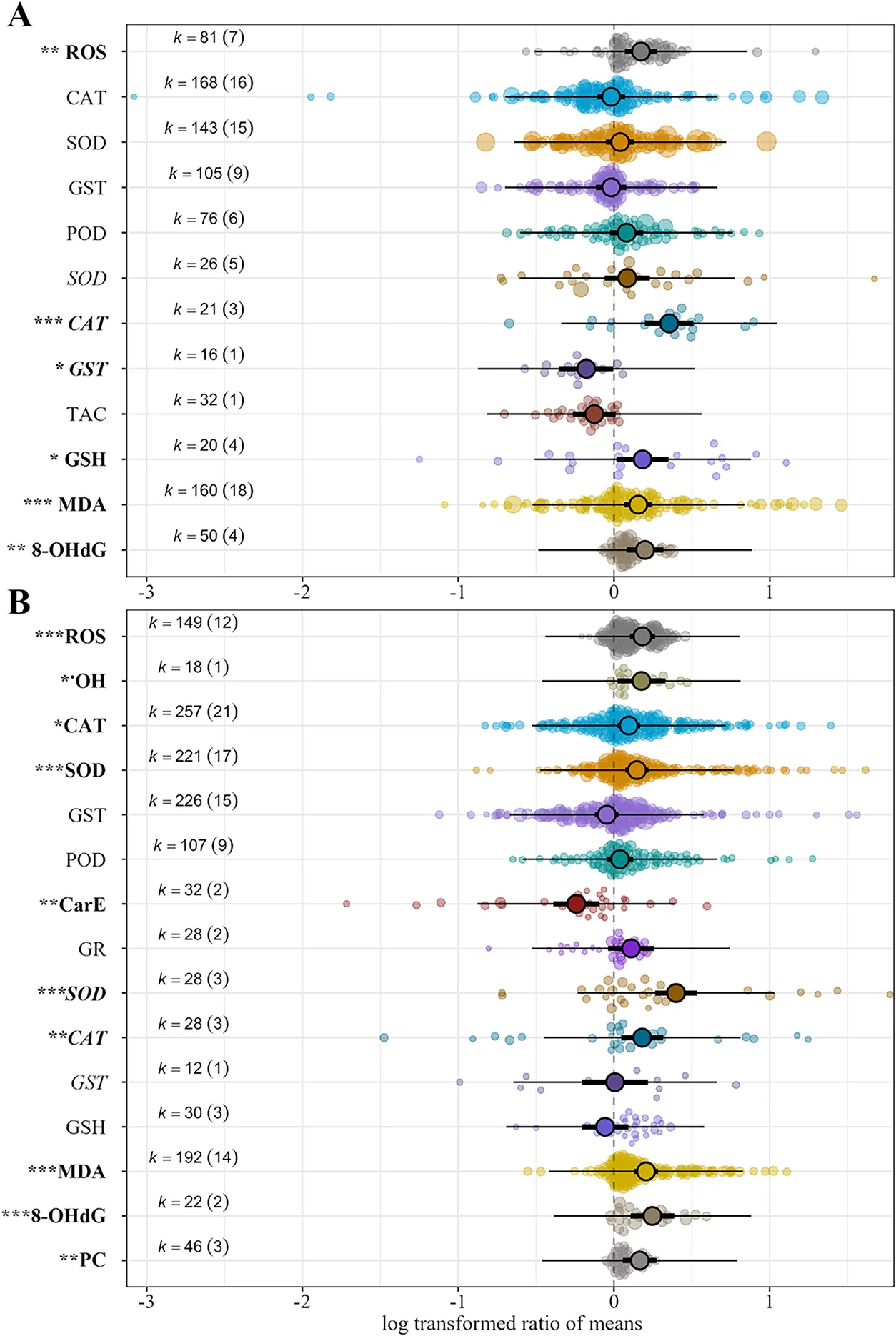
Nano- and microplastic particles (NMP) and neonicotinoids (neonics) induce the formation of reactive oxygen species (ROS) and alter oxidative stress responses on average, but with large variances. Overall effects of NMP (A) and neonics (B) on the generation of ROS, antioxidant enzyme activities and expression, and oxidative stress-associated damage products. K: number of data points (number of studies in brackets). The black circle displays the mean, thicker black lines show 95% confidence intervals, and narrow lines show prediction intervals. These estimates were calculated using mixed meta regression models without an intercept for each pollutant separately. Point sizes correlate with inverse standard errors. Oxidative stress markers in bold significantly differ from zero (no effect); *P < 0.05, **P < 0.01, ***P < 0.001. Italic oxidative stress markers represent mRNA expression levels of respective enzymes. ROS: reactive oxygen species concentration; ^•^OH: hydroxyl concentration; CAT: catalase activity; SOD: superoxide dismutase activity; GST: glutathione-S-transferase activity; POD: peroxidase activity; CarE: carboxylesterase activity; GR: glutathione reductase activity; TAC: total antioxidant capacity; GSH: glutathione concentration; MDA: malondialdehyde concentration; 8-OHdG: 8-hydroxy-2-deoxyguanosine concentration; and PC: protein carbonyl group concentration.

### 3.2 Sample Sizes Assuming a General Stress Response

We observed large variances and a frequent overlap of observed data ranges with zero (Figure 1) as well as small sample sizes in the compiled literature (maximum sample size: *N* = 8, median: *N* = 3). Small effect size, high variance, and low sample size decrease statistical power. Therefore, our findings raise the question of whether the sample sizes of experiments commonly conducted in individual studies are large enough to provide sufficient statistical power.

To investigate statistical power in more detail, we conducted simulation experiments and power analyses based on the means and standard deviations of measured effects for the control (without pollutant) and the treatment group (with pollutant) (see Section 2.3). To this end, we assumed the difference between the control and treatment means to be the true effects for each oxidative stress marker and pollutant group. For the sample sizes reported in the compiled literature (median of all 45 articles: *N* = 3), our simulations showed very low statistical power (Table S5) with an average power of 0.085 ± 0.102 (mean ± SD) and 0.065 ± 0.069 over all oxidative stress markers for NMP and neonics, respectively. This means that effects that are truly there would be detected in less than ten percent of the experiments. At a sample size of *N* = 8 (maximum sample size reported in the compiled literature), the average power for NMP and neonic oxidative stress markers was 0.220 ± 0.300 and 0.163 ± 0.229, respectively (Figures S4 and S5). Usually, a power of 0.8 is recommended for experimental design.^37,38^ We thus used additional simulations to investigate whether sufficient power could be achieved by increasing sample sizes up to a maximum of 100. Most oxidative stress markers (8 out of 12 and 9 out of 15 oxidative stress markers for NMP and neonics, respectively) did not reach a power of 80% with a sample size of 100 (Table S5). This indicates that, assuming a generalized ROS-related stress response (similar true effects across all NMP and neonics, respectively), the effects of exposure to NMP and neonics on oxidative stress markers are hardly detectable in single experimental studies.

### 3.3 Influence of Experimental Design and Pollution Properties

A potential explanation for the high data spread in the measured oxidative stress markers is a differential stress response, with effects being moderated by differences in the pollutants the organisms are exposed to or by variations resulting from differences in the experimental designs. An indication of such a differential stress response is the high residual heterogeneity we found in the meta-analytical models (test of residual heterogeneity: NMP: QE = 969,050.145, *P* < 0.001; neonics: QE = 153,342.470, *P* < 0.001). As a next step, we thus examined whether differences in effects are associated with different exposure designs, particle characteristics, and different neonicotinoid identities. Effects on the life-history traits of several soil-dwelling organisms have been shown to be dependent on NMP properties,^39^ while different neonics have different effects on the reproduction and survival of various soil animals at similar concentrations.^40^ As moderators of NMP effects, we considered the exposure time, concentration, particle shape, particle size, and polymer type in our analysis. As moderators of neonic effects, we included the exposure time, concentration, and neonic identity.

We used two approaches to examine whether each property contributed to the observed variance. First, we investigated differences among different pollutant properties and experimental parameters across all pooled oxidative stress markers. To this end, we fitted a full model containing all moderators as fixed effects and used likelihood ratio tests (LRT) to compare this model to different reduced models, where one moderator at a time was excluded. For this analysis, we assumed that effects on different oxidative stress markers are similar. This assumption could be too simplistic, however, since a pollutant could have different effects on different oxidative stress markers. For example, higher NMP concentrations can increase damage product concentrations such as those of MDA but decrease the activities of multiple antioxidant enzymes, e.g., CAT, SOD, and GST.^41^ So, in a second approach, we additionally considered differences in effects among different oxidative stress markers. To this end, we included interaction terms for each moderator with the oxidative stress markers in the model structure. Again, we compared the full model with all interactions to reduced models, where one moderator-oxidative stress marker interaction at a time was excluded. In addition, we used the variance components σ^2^ of the models to calculate the amount of the variance in the data that can be explained by the pollutants’ properties and experimental design parameters.

For models without interactions, the experimental exposure time (LR = 16.276, *P* < 0.001) and the NMP properties polymer type (LR = 38.465, *P* < 0.001), shape (LR = 12.634, *P* = 0.027), and size (LR = 6.312, *P* = 0.010) explained a considerable amount of the variability, while concentration (LR = 2.023, *P* = 0.154) did not. In contrast, for neonics, only the neonic concentration (LR = 26.409, *P* < 0.001) explained a significant amount of the variability, while exposure time (LR = 0.090, *P* = 0.764) and the neonic identity (LR = 21.462, *P* = 0.161) did not.

When interaction terms were included, we found that different NMP concentrations (LR = 34.730, *P* < 0.001), exposure times (LR = 32.457, *P* < 0.001), and polymers (LR = 30.043, *P* = 0.026) had different effects among the oxidative stress markers, but different shapes (LR = 21.950, *P* = 0.234) and sizes (LR = 3.564, *P* = 0.468) did not. Similarly, different neonic concentrations (LR = 59.767, *P* < 0.001), exposure times (LR = 35.283, *P* = 0.001), and neonic identities (LR = 345.732, *P* < 0.001) differed in the patterns observed for different oxidative stress markers. These results indicate that pollutant properties and experimental design parameters explain at least part of the observed variance in our data (test of moderators of full model with interactions: NMP: QM_91_ = 427.725, *P* < 0.001; neonics: QM_127_ = 738.941, *P* < 0.001). In total, pollutant properties and experimental design parameters accounted for 46.7% (NMP data set) and 21.2% (neonic data set) of the variance in the data. An in-depth analysis of how experimental designs and pollutant properties affect individual oxidative stress markers is reported in Figures S6–S13 and Tables S6–S13.

Our results indicate that ROS responses are strongly dependent on the pollutant properties, their chemical identities, and experimental design parameters. This complicates generalizations and predictions of the effects of pollutants on oxidative stress markers. Interestingly, only the neonic concentration, but not the NMP concentration, explained a significant amount of the variance in our data. This is surprising since other meta-analyses found concentration-dependent effects of NMP on the survival, growth rate, and reproduction of soil biota.^36,42^ However, our analyses revealed significant interactions of both pollutant properties (e.g., NMP concentration) and experimental design parameters with oxidative stress markers, indicating that the effects of these parameters act more strongly on certain oxidative stress markers than on others (see Figures S6–S13). In addition, pollutant parameters may interact not only with oxidative stress markers but also with each other, potentially leading to additional confounding effects. A more in-depth mechanistic understanding is thus needed to explain these differences and allow the extrapolation from tested pollutant-oxidative stress marker combinations to untested ones.

We hypothesize that the remaining unexplained variance mostly comes from unmeasured and/or unreported pollutant properties (such as surface charge of NMP), different measurement techniques and instruments, and genetic differences, even within the same species. However, how the reported pollutant properties and experimental designs affect ROS formation, subsequent damage product formation, and ROS responses is still unclear. Neonics seem to induce ROS generation by disrupting intracellular Ca^2+^ homeostasis,^21^ but why different neonics differ in their effects on oxidative stress markers remains understudied. For NMP, few hypotheses have been formulated on how these particles can induce oxidative stress, and our knowledge about the mediating effects remains fragmentary to date. For instance, NMP can induce mitochondrial ROS, which potentially exacerbates mitochondrial dysfunction.^43^ Qiao and colleagues suggested a general activation of the immune system, which in turn may lead to increased ROS responses,^16^ while Hu and Palić hypothesized that ROS formed during the environmental degradation of NMP might be taken up by organisms together with the particles and thus lead to higher ROS levels in these organisms.^44^ In addition, surface modifications of NMP, including surface morphology and surface charge, were shown to significantly affect particle-immune cell interactions,^45,46^ which might give a first insight into the moderating effects of these particle properties on ROS-related responses. Further research investigating the mechanistic pathways leading to and moderating ROS responses will be valuable to get a better understanding of the observed patterns.

### 3.4 Sample Sizes Depending on Properties

Since the pollutant-induced effects on ROS generation, ROS responses, and damage product formation seem to depend on the experimental design and partially on the pollutants’ properties, our first power analysis (which assumed a generalized ROS response) was likely too simplistic and may have overestimated the sample variance and consequently underestimated the statistical power of the test system. We thus performed more detailed data simulations and power analyses that considered differences in effects based on pollutant properties and experimental designs. To this end, we calculated the means and standard deviations of the measured oxidative stress markers for each combination of moderators (NMP: exposure duration, concentration, polymer, size, shape (813 unique combinations); neonic: exposure duration, concentration, neonic identity (1241 unique combinations)). These values were used as a basis for extensive data simulations and the calculation of statistical power dependent on sample size for each combination separately. As expected, for most oxidative stress markers, the statistical power at fixed sample sizes was notably higher when all moderators were considered. All 12 and 9 out of 15 oxidative stress markers needed sample sizes smaller than 20 to reach a power of at least 0.8 for NMP and neonics, respectively (Table 2). Seven NMP and 1 neonic oxidative stress marker required sample sizes smaller than ten. For example, based on our simulations, sample sizes of *N* = 3 and *N* = 5 are sufficient to achieve significant results in over 80% of experiments when measuring CAT or SOD mRNA expression after NMP exposure (Figure S14). For neonics, only the CarE activity, GR activity, and GSH concentration showed rather low power, not reaching the 0.8 threshold even with a sample size of *N* ≥ 50 (Figure S15).

**Table 2:** Most Oxidative Stress Measurements Have High Enough Power at Sample Sizes Lower than 20^a^

	NMP			neonics		
oxidative stress marker	smallest *N* with power ≥0.8	power at *N* = 3	power at *N* = 8	smallest *N* with power ≥0.8	power at *N* = 3	power at *N* = 8
ROS concentration	12	0.57	0.76	11	0.63	0.78
^•^OH concentration	NA	NA	NA	10	0.44	0.61
CAT activity	16	0.56	0.73	14	0.51	0.74
SOD activity	8	0.63	0.8	12	0.51	0.75
GST activity	9	0.59	0.79	24	0.53	0.7
POD activity	12	0.55	0.76	23	0.42	0.66
CarE activity	NA	NA	NA	>50	0.48	0.62
GR activity	NA	NA	NA	>50	0.26	0.45
SOD mRNA expression	5	0.69	0.89	10	0.59	0.79
CAT mRNA expression	3	0.88	0.91	17	0.63	0.73
GST mRNA expression	6	0.61	0.85	13	0.45	0.71
TAC	15	0.54	0.77	NA	NA	NA
GSH concentration	9	0.63	0.78	>50	0.19	0.41
MDA concentration	15	0.52	0.75	13	0.56	0.76
8-OHdG concentration	5	0.69	0.87	8	0.62	0.82
PC concentration	NA	NA	NA	25	0.38	0.61

a Smallest sample size (*N*) needed to achieve statistical power of at least 0.8 and statistical power of oxidative stress markers at *N* = 3 (most used sample size) and *N* = 8 (highest used sample size) after nano- and microplastic (NMP) or neonicotinoid (neonic) exposure in experiments investigating pollutant effects on oxidative stress markers in annelid worms. Means and standard deviations for each property and experimental design combination were used as a basis for data and power simulations. NA: no data on this oxidative stress marker available. ROS: reactive oxygen species concentration; ^•^OH: hydroxyl concentration; CAT: catalase activity; SOD: superoxide dismutase activity; GST: glutathione-S-transferase activity; POD: peroxidase activity; CarE: carboxylesterase activity; GR: glutathione reductase activity; TAC: total antioxidant capacity; GSH: glutathione concentration; MDA: malondialdehyde concentration; 8-OHdG: 8-hydroxy-2-deoxyguanosine concentration; and PC: protein carbonyl group concentration.

Statistical power depends on the sample size, true effect size, type I error rate (i.e., chosen α level), and the variance of the data.^13^ While the true effect size is fixed by the studied system and the type I error rate is usually fixed by the research community, the sample size and variance can be at least partially adjusted. Considering differential oxidative stress responses dependent on differences in NMP properties and neonic identities, the effects of pollutants on ROS generation, antioxidant responses, and damage products can likely be measured reliably with reasonably large sample sizes (at least *N* = 5 and *N* = 10 for most oxidative stress markers in NMPs and neonics, respectively). To further increase power, the variance in the data could be decreased by reducing the measurement error. For example, Murphy and colleagues argued that measurement techniques frequently used for measuring oxidative stress markers are often not optimal.^1^ Using an enzyme-linked immunosorbent assay (ELISA) to measure 8-OHdG concentrations^47^ or MDA to examine lipid peroxidation^48^ lacks specificity compared to more sophisticated methods like UPLC-MS/MS. Our data indicate that expression measurements via qPCR require smaller sample sizes compared to other oxidative stress markers, especially after NMP exposure. Improved methodology could thus help to strengthen statistical power, where adequate sample sizes are difficult to achieve.

We investigated how NMP and neonics affect oxidative stress markers in terrestrial annelids. However, caution should be taken when applying our insights to other organisms in other ecosystems. Since the underlying mechanisms of how these pollutants induce ROS are not well understood, especially for NMP, it is difficult to estimate how well our results can be generalized to other organisms. For example, if ROS are primarily produced by immune cells in response to pollutant exposure, the effects of these pollutants could differ greatly between organisms with different immune systems, such as annelids and vertebrates. On the other hand, property-dependent effects have been observed in other organisms as well, e.g., polymer-dependent effects on bacterial and fungal diversity.^42^ Therefore, the influence of pollutant properties and experimental design on oxidative stress markers probably applies to other taxa as well. While we have mainly analyzed the effects of pristine NMP, we have not considered the presence of additives or the numerous changes to NMP due to environmental factors. Plastic materials contain, on average, about 20 additives, including, among other antioxidants, plasticizers, and flame retardants,^49^ which can have additional adverse effects.^16^ Moreover, abiotic factors such as photooxidation via sunlight can lead to changes in the particles’ surface structure and increase the number of functional groups on the surface,^50^ which could explain the greater toxicity of aged particles compared to pristine NMP in some studies.^51^ Finally, the scarce mechanistic understanding of how pollutants influence individual oxidative stress markers limits the ability to test for antagonistic and synergistic effects among oxidative stress markers. For example, it is uncertain whether the increase in CAT and SOD activities ultimately led to lower amounts of MDA, 8-OHdG, and PC after neonic exposure, since these enzymes potentially decomposed ROS before they reacted and produced new damage products.

On the basis of these results, we identified three major improvements for future studies when analyzing the impacts of pollutants on oxidative stress markers. First, authors should report their methods (e.g., the specific oxidative stress markers, experimental design choices, and pollutant properties) with as much detail as possible.^52^ If possible, the materials, methods, and results should additionally be reported in a machine-readable format and uploaded to an online data repository that follows the FAIR principles (e.g., Zenodo, Open Science Framework) and additionally published as online Supporting Information

Second, to understand how ROS responses are moderated by pollutant properties, a more detailed knowledge of the molecular mechanisms leading to these differential responses and further investigation of adverse outcome pathways are necessary. Dedicated experiments are needed to clarify the mechanisms by which pollutants trigger effects.

Third, caution should be exercised when interpreting studies that use small sample sizes and suboptimal measurement techniques, since they most likely possess insufficient statistical power. Instead, more emphasis should be placed on increasing the statistical power of individual experiments by increasing the sample size, using more precise measurement techniques such as qPCR or LCMS, or both. On the basis of the data we compiled, we believe that a minimum of five replicates for NMP and ten replicates for neonic studies seem to be necessary to achieve sufficient power. Ideally, a priori power analyses based on lab-specific experimental conditions and variance estimates derived from previous experiments should be conducted to ensure that sample sizes meet statistical power requirements (i.e., a statistical power of at least 80%). This way, true effects can be detected at higher rates, and true effect sizes and potential risks to environmental health can be estimated more accurately.

## Supplementary Material



## Data Availability

All data and code pertaining to this manuscript are available on Zenodo https://zenodo.org/records/21390545 and GitHub (https://github.com/StatEcotox/Doering-et-al-2026).

## Notes

Additional regression tables (Tables S1–S4 and S6–S13), a power analysis table (Table S5), the analysis of contact tests (Figure S1), the PRISMA Flow diagram (Figure S2), a plot displaying the results of a reanalysis including the 20 values with sampling variance of 10^–7^ instead of zero (Figure S3), power analysis plots (Figures S4, S5, S14, and S15), and pollutant property and experimental design specific figures (Figures S6–S13). (DOCX)
