# Supplementary material for: Oxidative Stress after Pollutant Exposure Depends Strongly on Experimental Design and Pollutant Properties in Annelids: A Meta-Analysis
Source: Environ Sci Technol. 2026 Aug 13;60(33):23124–33. doi: 10.1021/acs.est.6c03847 (PMC13524028; doi:10.1021/acs.est.6c03847)
Supplement: Supplementary file 1 [file es6c03847_si_001.docx]

**Supporting information**

**Oxidative stress after pollutant exposure depends strongly on experimental design and pollutant properties in annelids: a meta-analysis**

Max V.R. Döring^1^, Heike Feldhaar^1,2^, Ana L. Antonio Vital^3^, Magdalena M. Mair^2,3,*^

^1^ Animal Population Ecology, Animal Ecology I, University of Bayreuth, 95440 Bayreuth, Germany

^2^ Bayreuth Centre of Ecology and Environmental Research (BayCEER), 95440 Bayreuth, Germany

^3^ Statistical Ecotoxicology, University of Bayreuth, 95440 Bayreuth, Germany

^*^ Corresponding author

Correspondence to: magdalena.mair@uni-bayreuth.de

Number of pages: 30

Number of tables: 13

Number of figures: 15

**Supplementary Table S1. Regression table of the overall effects of neonicotinoids on the generation of reactive oxygen species (ROS), antioxidant enzyme activities and expression, and oxidative stress-associated damage products, determined via contact tests.** ROS: reactive oxygen species concentration; CAT: catalase activity; GST: glutathione S-transferase activity; GSH: glutathione concentration; GR: glutathione reductase activity; CarE: Carboxylesterase activity; MDA: malondialdehyde concentration.

| oxidative stress markers | number of studies | estimate | standard error | standard deviation | CI lower bound | CI upper bound | Z value | *P* value |
| --- | --- | --- | --- | --- | --- | --- | --- | --- |
| ROS | 2 | 0.2676 | 0.0961 | 0.4734 | 0.0793 | 0.4559 | 2.7860 | 0.0053 |
| CAT | 3 | 0.0754 | 0.0776 | 0.2358 | -0.0766 | 0.2274 | 0.9728 | 0.3306 |
| GST | 3 | 0.0415 | 0.0798 | 0.4237 | -0.1148 | 0.1979 | 0.5206 | 0.6027 |
| CarE | 1 | 0.1455 | 0.0975 | 0.0934 | -0.0457 | 0.3366 | 1.4918 | 0.1358 |
| GR | 1 | 0.1521 | 0.0992 | 0.0886 | -0.0424 | 0.3466 | 1.5326 | 0.1254 |
| GSH | 3 | -0.0407 | 0.0791 | 0.3001 | -0.1958 | 0.1143 | -0.5147 | 0.6067 |
| MDA | 1 | 0.5002 | 0.1816 | 0.2211 | 0.1442 | 0.8562 | 2.7541 | 0.0059 |

**Supplementary Table S2. Regression table of overall nano- and microplastic particles (NMP) effects including 20 values where sampling variances of zero were substituted by values of 10^-7^ instead of excluding them from the analysis**. Italic oxidative stress markers represent mRNA expression level of the respective enzyme. CI: 95% confidence interval; ROS: reactive oxygen species concentration; CAT: catalase activity; SOD: superoxide dismutase activity; GST: glutathione-S-transferase activity; POD: peroxidase activity; TAC: total antioxidant capacity; GSH: glutathione concentration; MDA: malondialdehyde concentration; 8-OHdG: [8-hydroxy-2-deoxyguanosine](https://www.ncbi.nlm.nih.gov/pmc/articles/PMC3261525/) concentration.

| oxidative stress markers | number of studies | estimate | standard error | standard deviation | CI lower bound | CI upper bound | Z value | *P* value |
| --- | --- | --- | --- | --- | --- | --- | --- | --- |
| ROS | 7 | 0.1734 | 0.0530 | 0.2462 | 0.0695 | 0.2773 | 3.2719 | 0.0011 |
| CAT | 16 | -0.0198 | 0.0454 | 0.4542 | -0.1089 | 0.0693 | -0.4356 | 0.6631 |
| SOD | 15 | 0.0466 | 0.0462 | 0.2698 | -0.0440 | 0.1371 | 1.0078 | 0.3135 |
| GST | 9 | -0.0190 | 0.0501 | 0.2552 | -0.1172 | 0.0792 | -0.3794 | 0.7044 |
| POD | 6 | 0.0779 | 0.0546 | 0.3282 | -0.0291 | 0.1848 | 1.4274 | 0.1535 |
| *SOD* | 5 | 0.0863 | 0.0742 | 0.5288 | -0.0592 | 0.2317 | 1.1627 | 0.2450 |
| *CAT* | 3 | 0.3546 | 0.0800 | 0.3501 | 0.1978 | 0.5114 | 4.4327 | <.0001 |
| *GST* | 1 | -0.1774 | 0.0894 | 0.1653 | -0.3526 | -0.0021 | -1.9831 | 0.0474 |
| TAC | 1 | -0.1259 | 0.0707 | 0.1671 | -0.2644 | 0.0127 | -1.7808 | 0.0749 |
| GSH | 4 | 0.1843 | 0.0857 | 0.5912 | 0.0164 | 0.3523 | 2.1508 | 0.0315 |
| MDA | 18 | 0.1545 | 0.0450 | 0.3890 | 0.0664 | 0.2426 | 3.4368 | 0.0006 |
| 8-OHdG | 4 | 0.1995 | 0.0607 | 0.1050 | 0.0806 | 0.3184 | 3.2894 | 0.0010 |

**Supplementary Table S3. Regression table of overall nano- and microplastic particles (NMP) effects**. Italic oxidative stress markers represent mRNA expression level of the respective enzyme. CI: 95% confidence interval; ROS: reactive oxygen species concentration; CAT: catalase activity; SOD: superoxide dismutase activity; GST: glutathione-S-transferase activity; POD: peroxidase activity; TAC: total antioxidant capacity; GSH: glutathione concentration; MDA: malondialdehyde concentration; 8-OHdG: [8-hydroxy-2-deoxyguanosine](https://www.ncbi.nlm.nih.gov/pmc/articles/PMC3261525/) concentration.

| oxidative stress markers | number of studies | estimate | standard error | standard deviation | CI lower bound | CI upper bound | Z value | *P* value |
| --- | --- | --- | --- | --- | --- | --- | --- | --- |
| ROS | 7 | 0.1733 | 0.0531 | 0.2462 | 0.0693 | 0.2773 | 3.2652 | 0.0011 |
| CAT | 16 | -0.0182 | 0.0457 | 0.4542 | -0.1077 | 0.0713 | -0.3986 | 0.6902 |
| SOD | 15 | 0.0399 | 0.0466 | 0.2716 | -0.0515 | 0.1313 | 0.8559 | 0.3920 |
| GST | 9 | -0.0174 | 0.0502 | 0.2552 | -0.1158 | 0.0811 | -0.3460 | 0.7294 |
| POD | 6 | 0.0812 | 0.0548 | 0.3282 | -0.0261 | 0.1886 | 1.4826 | 0.1382 |
| *SOD* | 5 | 0.0851 | 0.0740 | 0.5288 | -0.0598 | 0.2301 | 1.1509 | 0.2498 |
| *CAT* | 3 | 0.3542 | 0.0797 | 0.3501 | 0.1980 | 0.5104 | 4.4441 | <.0001 |
| *GST* | 1 | -0.1777 | 0.0890 | 0.1653 | -0.3522 | -0.0032 | -1.9956 | 0.0460 |
| TAC | 1 | -0.1262 | 0.0705 | 0.1671 | -0.2644 | 0.0120 | -1.7903 | 0.0734 |
| GSH | 4 | 0.1838 | 0.0854 | 0.5912 | 0.0164 | 0.3512 | 2.1514 | 0.0314 |
| MDA | 18 | 0.1563 | 0.0456 | 0.3846 | 0.0669 | 0.2457 | 3.4265 | 0.0006 |
| 8-OHdG | 4 | 0.1993 | 0.0606 | 0.1050 | 0.0806 | 0.3181 | 3.2895 | 0.0010 |

**Supplementary Table S4. Regression table of overall neonicotinoid (neonic) effects.** Italic oxidative stress markers represent mRNA expression level of the respective enzyme. CI: 95% confidence interval; ROS: reactive oxygen species concentration; ^•^OH: hydroxyl concentration; CAT: catalase activity; SOD: superoxide dismutase activity; GST: glutathione-S-transferase activity; POD: peroxidase activity; CarE: carboxylesterase activity; GR: glutathione reductase activity; GSH: glutathione concentration; MDA: malondialdehyde concentration; 8-OHdG: [8-hydroxy-2-deoxyguanosine](https://www.ncbi.nlm.nih.gov/pmc/articles/PMC3261525/) concentration; PC: protein carbonyl group concentration.

| oxidative stress markers | number of studies | estimate | standard error | standard deviation | CI lower bound | CI upper bound | Z value | *P* value |
| --- | --- | --- | --- | --- | --- | --- | --- | --- |
| ROS | 11 | 0.1834 | 0.0411 | 0.1299 | 0.1028 | 0.2640 | 4.4619 | <.0001 |
| ^•^OH | 1 | 0.1769 | 0.0788 | 0.1565 | 0.0224 | 0.3314 | 2.2435 | 0.0249 |
| CAT | 21 | 0.0955 | 0.0375 | 0.3477 | 0.0221 | 0.1690 | 2.5483 | 0.0108 |
| SOD | 17 | 0.1479 | 0.0385 | 0.3534 | 0.0724 | 0.2235 | 3.8371 | 0.0001 |
| GST | 15 | -0.0457 | 0.0390 | 0.3553 | -0.1221 | 0.0307 | -1.1735 | 0.2406 |
| POD | 9 | 0.0391 | 0.0441 | 0.3237 | -0.0474 | 0.1256 | 0.8858 | 0.3757 |
| CarE | 2 | -0.2410 | 0.0760 | 0.4659 | -0.3900 | -0.0921 | -3.1725 | 0.0015 |
| GR | 2 | 0.1102 | 0.0753 | 0.2324 | -0.0374 | 0.2577 | 1.4632 | 0.1434 |
| *SOD* | 3 | 0.3987 | 0.0689 | 0.5935 | 0.2638 | 0.5337 | 5.7904 | <.0001 |
| *CAT* | 3 | 0.1820 | 0.0687 | 0.5953 | 0.0473 | 0.3167 | 2.6475 | 0.0081 |
| *GST* | 1 | 0.0071 | 0.1085 | 0.5195 | -0.2056 | 0.2199 | 0.0658 | 0.9475 |
| GSH | 3 | -0.0564 | 0.0766 | 0.2174 | -0.2066 | 0.0937 | -0.7367 | 0.4613 |
| MDA | 14 | 0.2066 | 0.0394 | 0.2503 | 0.1293 | 0.2839 | 5.2406 | <.0001 |
| 8-OHdG | 2 | 0.2471 | 0.0720 | 0.1897 | 0.1060 | 0.3882 | 3.4317 | 0.0006 |
| PC | 3 | 0.1660 | 0.0554 | 0.0791 | 0.0573 | 0.2746 | 2.9938 | 0.0028 |

**Supplementary Table S5.** **Smallest sample size (N) where the statistical power exceeds 0.8 and statistical power of oxidative stress markers at N = 100 after nano- and microplastic particles (NMP) or neonicotinoid (neonics) exposure.** We used the mean and standard deviation of each oxidative stress marker as our underlying distributions. NA indicate unmeasured oxidative stress markers. ROS: reactive oxygen species concentration; ^•^OH: hydroxyl concentration; CAT: catalase activity; SOD: superoxide dismutase activity; GST: glutathione-S-transferase activity; POD: peroxidase activity; CarE: carboxylesterase activity; GR: glutathione reductase activity; TAC: total antioxidant capacity; GSH: glutathione concentration; MDA: malondialdehyde concentration; 8-OHdG: [8-hydroxy-2-deoxyguanosine](https://www.ncbi.nlm.nih.gov/pmc/articles/PMC3261525/) concentration; PC: protein carbonyl group concentration.

|  |  | NMP | | | neonics | | |
| --- | --- | --- | --- | --- | --- | --- | --- |
| oxidative stress marker | | smallest N with power ≥ 0.8 | power at N = 3 | power at N = 8 | smallest N with power ≥ 0.8 | power at N = 3 | power at N = 8 |
| ROS concentration | | >100 | 0.08 | 0.08 | >100 | 0.03 | 0.05 |
| ^•^OH radical levels | | NA | NA | NA | 11 | 0.24 | 0.66 |
| CAT activity | | >100 | 0.01 | 0.05 | >100 | 0.03 | 0.09 |
| SOD activity | | >100 | 0.04 | 0.06 | >100 | 0.03 | 0.04 |
| GST activity | | >100 | 0.02 | 0.04 | >100 | 0.05 | 0.07 |
| POD activity | | >100 | 0.04 | 0.02 | >100 | 0.03 | 0.00 |
| CarE activity | | NA | NA | NA | 94 | 0.01 | 0.07 |
| GR activity | | NA | NA | NA | >100 | 0.03 | 0.04 |
| SOD mRNA expression | | 83 | 0.07 | 0.09 | 28 | 0.16 | 0.23 |
| CAT mRNA expression | | 10 | 0.14 | 0.60 | 60 | 0.07 | 0.14 |
| GST mRNA expression | | 7 | 0.38 | 0.91 | >100 | 0.02 | 0.08 |
| TAC | | 13 | 0.13 | 0.59 | NA | NA | NA |
| GSH concentration | | >100 | 0.03 | 0.08 | 97 | 0.03 | 0.05 |
| MDA concentration | | >100 | 0.04 | 0.08 | >100 | 0.05 | 0.04 |
| 8-OHdG concentration | | >100 | 0.04 | 0.04 | 11 | 0.18 | 0.76 |
| PC concentration | | NA | NA | NA | >100 | 0.04 | 0.12 |

**Supplementary Table S6. Regression table of nano- and microplastic particles (NMP) effects over time.** Italic oxidative stress markers represent mRNA expression level of the respective enzyme. The GST mRNA expression level was modelled only with a linear term. CI: 95% confidence interval; ROS: reactive oxygen species concentration; CAT: catalase activity; SOD: superoxide dismutase activity; GST: glutathione-S-transferase activity; POD: peroxidase activity; TAC: total antioxidant capacity; GSH: glutathione concentration; MDA: malondialdehyde concentration; 8-OHdG: [8-hydroxy-2-deoxyguanosine](https://www.ncbi.nlm.nih.gov/pmc/articles/PMC3261525/) concentration.

| oxidative stress marker | term | estimate | standard error | CI lower bound | CI upper bound | Z value | P value |
| --- | --- | --- | --- | --- | --- | --- | --- |
| ROS | linear | -0.0169 | 0.0107 | -0.0378 | 0.004 | -1.5828 | 0.1135 |
|  | quadratic | 0.0004 | 0.0003 | -0.0001 | 0.0009 | 1.5229 | 0.1278 |
| CAT | linear | -0.0139 | 0.0094 | -0.0323 | 0.0044 | -1.4905 | 0.1361 |
|  | quadratic | 0.0003 | 0.0002 | 0.0000 | 0.0007 | 1.8923 | 0.0584 |
| SOD | linear | -0.0094 | 0.0078 | -0.0247 | 0.0058 | -1.2087 | 0.2268 |
|  | quadratic | 0.0002 | 0.0002 | -0.0001 | 0.0006 | 1.3611 | 0.1735 |
| GST | linear | -0.0698 | 0.0142 | -0.0976 | -0.0420 | -4.9265 | <.0001 |
|  | quadratic | 0.0017 | 0.0004 | 0.0009 | 0.0025 | 4.3872 | <.0001 |
| POD | linear | -0.0794 | 0.0236 | -0.1256 | -0.0333 | -3.3728 | 0.0007 |
|  | quadratic | 0.0022 | 0.0007 | 0.0009 | 0.0034 | 3.284 | 0.0010 |
| SOD | linear | -0.2238 | 0.0456 | -0.3132 | -0.1344 | -4.9064 | <.0001 |
|  | quadratic | 0.0044 | 0.0011 | 0.0022 | 0.0065 | 3.9219 | <.0001 |
| CAT | linear | -0.3336 | 0.0498 | -0.4313 | -0.2360 | -6.6974 | <.0001 |
|  | quadratic | 0.0082 | 0.0012 | 0.0058 | 0.0106 | 6.6594 | <.0001 |
| GST | linear | -0.0116 | 0.0052 | -0.0218 | -0.0013 | -0.1493 | 0.0271 |
| TAC | linear | 0.0049 | 0.0189 | -0.0321 | 0.0419 | 0.2587 | 0.7959 |
|  | quadratic | -0.0004 | 0.0005 | -0.0015 | 0.0006 | -0.8334 | 0.4046 |
| GSH | linear | -0.1721 | 0.0885 | -0.3455 | 0.0013 | -1.9457 | 0.0517 |
|  | quadratic | 0.004 | 0.0026 | -0.0011 | 0.0090 | 1.5489 | 0.1214 |
| MDA | linear | -0.0461 | 0.0143 | -0.074 | -0.0182 | -3.2344 | 0.0012 |
|  | quadratic | 0.0011 | 0.0004 | 0.0004 | 0.0018 | 2.9339 | 0.0033 |
| 8-OhdG | linear | -0.0191 | 0.0085 | -0.0358 | -0.0024 | -2.2392 | 0.0251 |
|  | quadratic | 0.0007 | 0.0002 | 0.0003 | 0.0012 | 3.1228 | 0.0018 |

**Supplementary Table S7. Regression table of nano- and microplastic particles (NMP) effects at different concentrations.** Italic oxidative stress markers represent mRNA expression level of the respective enzyme. The GSH concentration and GST mRNA expression level were modelled only with a linear term. CI: 95% confidence interval; ROS: reactive oxygen species concentration; CAT: catalase activity; SOD: superoxide dismutase activity; GST: glutathione-S-transferase activity; POD: peroxidase activity; TAC: total antioxidant capacity; GSH: glutathione concentration; MDA: malondialdehyde concentration; 8-OHdG: [8-hydroxy-2-deoxyguanosine](https://www.ncbi.nlm.nih.gov/pmc/articles/PMC3261525/) concentration.

| oxidative stress marker | term | estimate | standard error | CI lower bound | CI upper bound | Z value | P value |
| --- | --- | --- | --- | --- | --- | --- | --- |
| ROS | linear | 0.0113 | 0.0313 | -0.0502 | 0.0727 | 0.3598 | 0.7190 |
|  | quadratic | -0.0064 | 0.0075 | -0.0211 | 0.0083 | -0.855 | 0.3925 |
| CAT | linear | 0.0798 | 0.0303 | 0.0203 | 0.1392 | 2.6299 | 0.0085 |
|  | quadratic | 0.0156 | 0.0086 | -0.0013 | 0.0325 | 1.8068 | 0.0708 |
| SOD | linear | -0.0238 | 0.0226 | -0.0682 | 0.0206 | -1.0512 | 0.2931 |
|  | quadratic | -0.0036 | 0.0027 | -0.009 | 0.0018 | -1.3109 | 0.1899 |
| GST | linear | -0.0519 | 0.0238 | -0.0987 | -0.0052 | -2.1800 | 0.0293 |
|  | quadratic | -0.0077 | 0.0061 | -0.0197 | 0.0042 | -1.2662 | 0.2054 |
| POD | linear | 0.0560 | 0.0306 | -0.004 | 0.1161 | 1.8282 | 0.0675 |
|  | quadratic | 0.0178 | 0.0086 | 0.0009 | 0.0348 | 2.0609 | 0.0393 |
| SOD | linear | -0.2073 | 0.4665 | -1.1217 | 0.7071 | -0.4443 | 0.6568 |
|  | quadratic | -0.0115 | 0.1239 | -0.2543 | 0.2312 | -0.0931 | 0.9258 |
| CAT | linear | -0.4375 | 0.3071 | -1.0394 | 0.1644 | -1.4245 | 0.1543 |
|  | quadratic | -0.1253 | 0.0863 | -0.2944 | 0.0438 | -1.4524 | 0.1464 |
| GST | linear | -0.3378 | 0.7951 | -1.8962 | 1.2207 | -0.4248 | 0.6710 |
|  | quadratic | -0.0326 | 0.1704 | -0.3666 | 0.3013 | -0.1916 | 0.8481 |
| TAC | linear | -1.1331 | 0.4878 | -2.0893 | -0.1769 | -2.3227 | 0.0202 |
|  | quadratic | -0.1988 | 0.1044 | -0.4036 | 0.0059 | -1.9039 | 0.0569 |
| GSH | linear | 0.1286 | 0.0619 | 0.0073 | 0.2500 | 2.0772 | 0.0378 |
|  | quadratic | 0.0165 | 0.0066 | 0.0034 | 0.0295 | 2.4761 | 0.0133 |
| MDA | linear | -0.0191 | 0.0341 | -0.0860 | 0.0478 | -0.5598 | 0.5756 |
|  | quadratic | -0.0155 | 0.0078 | -0.0307 | -0.0002 | -1.9885 | 0.0468 |
| 8-OHdG | linear | -0.0204 | 0.0539 | -0.1260 | 0.0852 | -0.3785 | 0.7051 |
|  | quadratic | -0.0198 | 0.0155 | -0.0502 | 0.0107 | -1.2734 | 0.2029 |

**Supplementary Table S8. Regression table of nano- and microplastic particles (NMP) effects at different sizes.** Italic oxidative stress markers represent mRNA expression level of the respective enzyme. The effects of different NMP sizes on the TAC and GST mRNA expression could not be evaluated since only one size was used. CI: 95% confidence interval; ROS: reactive oxygen species concentration; CAT: catalase activity; SOD: superoxide dismutase activity; GST: glutathione-S-transferase activity; POD: peroxidase activity; TAC: total antioxidant capacity; GSH: glutathione concentration; MDA: malondialdehyde concentration; 8-OHdG: [8-hydroxy-2-deoxyguanosine](https://www.ncbi.nlm.nih.gov/pmc/articles/PMC3261525/) concentration.

| oxidative stress marker | term | estimate | standard error | CI lower bound | CI upper bound | Z value | P value |
| --- | --- | --- | --- | --- | --- | --- | --- |
| ROS | linear | 0.0283 | 0.0149 | -0.001 | 0.0576 | 1.8928 | 0.0584 |
|  | quadratic | -0.0008 | 0.0007 | -0.0023 | 0.0006 | -1.1004 | 0.2712 |
| CAT | linear | -0.0516 | 0.0312 | -0.1126 | 0.0095 | -1.6549 | 0.0979 |
|  | quadratic | 0.0024 | 0.0014 | -0.0004 | 0.0052 | 1.7092 | 0.0874 |
| SOD | linear | 0.0035 | 0.0259 | -0.0473 | 0.0543 | 0.1353 | 0.8923 |
|  | quadratic | -0.0002 | 0.0011 | -0.0024 | 0.0021 | -0.1446 | 0.885 |
| GST | linear | -0.0409 | 0.0333 | -0.1063 | 0.0244 | -1.2274 | 0.2197 |
|  | quadratic | 0.0018 | 0.0015 | -0.0011 | 0.0046 | 1.1994 | 0.2304 |
| POD | linear | 0.007 | 0.0235 | -0.0391 | 0.0532 | 0.2993 | 0.7647 |
|  | quadratic | -0.0005 | 0.0012 | -0.0029 | 0.0019 | -0.3927 | 0.6945 |
| SOD | linear | -0.2456 | 0.858 | -1.9273 | 1.436 | -0.2863 | 0.7747 |
|  | quadratic | 0.0113 | 0.0558 | -0.0981 | 0.1208 | 0.203 | 0.8391 |
| CAT | linear | -0.2825 | 0.3906 | -1.0482 | 0.4831 | -0.7233 | 0.4695 |
|  | quadratic | 0.0153 | 0.0249 | -0.0335 | 0.0641 | 0.6147 | 0.5388 |
| GSH | linear | -0.0259 | 0.1038 | -0.2293 | 0.1775 | -0.2494 | 0.8031 |
|  | quadratic | -0.0008 | 0.0039 | -0.0084 | 0.0068 | -0.197 | 0.8439 |
| MDA | linear | -0.0126 | 0.035 | -0.0812 | 0.0559 | -0.3609 | 0.7181 |
|  | quadratic | 0.0005 | 0.0015 | -0.0024 | 0.0034 | 0.3638 | 0.716 |
| 8-OHdG | linear | 0.0346 | 0.0128 | 0.0096 | 0.0597 | 2.7095 | 0.0067 |
|  | quadratic | -0.0016 | 0.0006 | -0.0029 | -0.0004 | -2.5827 | 0.0098 |

**Supplementary Table S9. Regression table of polystyrene (PS) and low-density polyethylene (LDPE) nano- and microplastic particles (NMP) on oxidative stress markers.** PS (497 data points) and LDPE (123 data points) are the two most used polymers in our data set. Italic oxidative stress markers represent mRNA expression level of the respective enzyme. CI: 95% confidence interval; ROS: reactive oxygen species concentration; CAT: catalase activity; SOD: superoxide dismutase activity; GST: glutathione-S-transferase activity; POD: peroxidase activity; TAC: total antioxidant capacity; GSH: glutathione concentration; MDA: malondialdehyde concentration; 8-OHdG: [8-hydroxy-2-deoxyguanosine](https://www.ncbi.nlm.nih.gov/pmc/articles/PMC3261525/) concentration.

| polymer | oxidative stress markers | estimate | standard error | CI lower bound | CI upper bound | Z value | *P* value |
| --- | --- | --- | --- | --- | --- | --- | --- |
| PS | ROS | 0.1088 | 0.0414 | 0.0276 | 0.1900 | 2.6260 | 0.0086 |
|  | CAT | -0.0223 | 0.0394 | -0.0997 | 0.0550 | -0.5661 | 0.5713 |
|  | SOD | 0.0169 | 0.0386 | -0.0588 | 0.0925 | 0.4368 | 0.6623 |
|  | GST | 0.0089 | 0.0415 | -0.0723 | 0.0902 | 0.2150 | 0.8297 |
|  | POD | 0.0734 | 0.0458 | -0.0164 | 0.1631 | 1.6017 | 0.1092 |
|  | *SOD* | 0.0824 | 0.0579 | -0.0310 | 0.1959 | 1.4242 | 0.1544 |
|  | *CAT* | 0.4315 | 0.0575 | 0.3189 | 0.5441 | 7.5091 | <.0001 |
|  | *GST* | -0.1974 | 0.0579 | -0.3110 | -0.0838 | -3.4066 | 0.0007 |
|  | TAC | -0.1456 | 0.0482 | -0.2400 | -0.0513 | -3.0245 | 0.0025 |
|  | GSH | 0.4837 | 0.0817 | 0.3237 | 0.6437 | 5.9236 | <.0001 |
|  | MDA | 0.1301 | 0.0396 | 0.0524 | 0.2078 | 3.2813 | 0.0010 |
|  | 8-OHdG | 0.1196 | 0.0477 | 0.0260 | 0.2132 | 2.5051 | 0.0122 |
| LDPE | ROS | 0.3386 | 0.3212 | -0.2910 | 0.9682 | 1.0540 | 0.2919 |
|  | CAT | -0.0796 | 0.0738 | -0.2242 | 0.0651 | -1.0781 | 0.2810 |
|  | SOD | -0.1770 | 0.1380 | -0.4474 | 0.0935 | -1.2826 | 0.1996 |
|  | GST | -0.1510 | 0.1116 | -0.3696 | 0.0677 | -1.3535 | 0.1759 |
|  | POD | 0.1487 | 0.1271 | -0.1003 | 0.3978 | 1.1705 | 0.2418 |
|  | MDA | 0.0562 | 0.0754 | -0.0916 | 0.2041 | 0.7454 | 0.4560 |
|  | 8-OHdG | 0.0858 | 0.3220 | -0.5452 | 0.7168 | 0.2665 | 0.7899 |

**Supplementary Table S10. Regression table of fragment and bead shaped nano- and microplastic particles (NMP) on oxidative stress markers.** Fragments (182 data points) and beads (161 data points) are the two most used shapes in our data set. Italic oxidative stress markers represent mRNA expression level of the respective enzyme. CI: 95% confidence interval; ROS: reactive oxygen species concentration; CAT: catalase activity; SOD: superoxide dismutase activity; GST: glutathione-S-transferase activity; POD: peroxidase activity; TAC: total antioxidant capacity; GSH: glutathione concentration; MDA: malondialdehyde concentration; 8-OHdG: [8-hydroxy-2-deoxyguanosine](https://www.ncbi.nlm.nih.gov/pmc/articles/PMC3261525/) concentration.

| shape | oxidative stress markers | estimate | standard error | CI lower bound | CI upper bound | Z value | *P* value |
| --- | --- | --- | --- | --- | --- | --- | --- |
| fragments | ROS | 0.1984 | 0.0559 | 0.0889 | 0.3079 | 3.5513 | 0.0004 |
|  | CAT | -0.2238 | 0.0555 | -0.3326 | -0.1150 | -4.0317 | <.0001 |
|  | SOD | -0.0731 | 0.0572 | -0.1852 | 0.0390 | -1.2777 | 0.2013 |
|  | GST | -0.1102 | 0.0559 | -0.2198 | -0.0007 | -1.9721 | 0.0486 |
|  | *SOD* | -0.0580 | 0.0793 | -0.2134 | 0.0975 | -0.7308 | 0.4649 |
|  | *CAT* | 0.4357 | 0.0785 | 0.2818 | 0.5896 | 5.5497 | <.0001 |
|  | *GST* | -0.3511 | 0.0790 | -0.5059 | -0.1962 | -4.4441 | <.0001 |
|  | TAC | -0.2639 | 0.0640 | -0.3893 | -0.1384 | -4.1224 | <.0001 |
|  | GSH | -0.5024 | 0.1077 | -0.7134 | -0.2913 | -4.6657 | <.0001 |
|  | MDA | 0.0873 | 0.0522 | -0.0150 | 0.1896 | 1.6733 | 0.0943 |
|  | 8-OHdG | 0.0728 | 0.0605 | -0.0457 | 0.1913 | 1.2038 | 0.2287 |
| beads | ROS | 0.0397 | 0.0299 | -0.0189 | 0.0982 | 1.3275 | 0.1844 |
|  | CAT | 0.0773 | 0.0286 | 0.0212 | 0.1334 | 2.6986 | 0.0070 |
|  | SOD | 0.1564 | 0.0257 | 0.1061 | 0.2068 | 6.0901 | <.0001 |
|  | GST | 0.0914 | 0.0307 | 0.0312 | 0.1516 | 2.9757 | 0.0029 |
|  | POD | 0.0427 | 0.0298 | -0.0157 | 0.1010 | 1.4340 | 0.1516 |
|  | MDA | 0.1603 | 0.0296 | 0.1023 | 0.2183 | 5.4165 | <.0001 |

**Supplementary Table S11. Regression table of neonicotinoids (neonics) effects over time.** Italic oxidative stress markers represent mRNA expression level of the respective enzyme. CI: 95% confidence interval; ROS: reactive oxygen species concentration; ^•^OH: hydroxyl concentration; CAT: catalase activity; SOD: superoxide dismutase activity; GST: glutathione-S-transferase activity; POD: peroxidase activity; CarE: carboxylesterase activity; GR: glutathione reductase activity; GSH: glutathione concentration; MDA: malondialdehyde concentration; 8-OHdG: [8-hydroxy-2-deoxyguanosine](https://www.ncbi.nlm.nih.gov/pmc/articles/PMC3261525/) concentration; PC: protein carbonyl group concentration.

| oxidative stress marker | term | estimate | standard error | CI lower bound | CI upper bound | Z value | P value |
| --- | --- | --- | --- | --- | --- | --- | --- |
| ROS | linear | 0.0021 | 0.0038 | -0.0053 | 0.0095 | 0.5523 | 0.5808 |
|  | quadratic | 0.0000 | 0.0001 | -0.0002 | 0.0002 | 0.0365 | 0.9709 |
| ^•^OH | linear | 0.0095 | 0.0073 | -0.0047 | 0.0238 | 1.3076 | 0.1910 |
|  | quadratic | -0.0002 | 0.0001 | -0.0005 | 0.0000 | -1.6897 | 0.0911 |
| CAT | linear | -0.0022 | 0.0042 | -0.0105 | 0.0061 | -0.5208 | 0.6025 |
|  | quadratic | 0.0000 | 0.0001 | -0.0001 | 0.0002 | 0.4327 | 0.6652 |
| SOD | linear | 0.0041 | 0.0049 | -0.0055 | 0.0137 | 0.8357 | 0.4033 |
|  | quadratic | 0.0000 | 0.0001 | -0.0002 | 0.0002 | -0.2968 | 0.7666 |
| GST | linear | -0.0105 | 0.0048 | -0.0200 | -0.0010 | -2.1680 | 0.0302 |
|  | quadratic | 0.0002 | 0.0001 | 0.0000 | 0.0004 | 1.9711 | 0.0487 |
| POD | linear | 0.0107 | 0.0118 | -0.0124 | 0.0338 | 0.9094 | 0.3632 |
|  | quadratic | -0.0005 | 0.0004 | -0.0012 | 0.0002 | -1.2950 | 0.1953 |
| CarE | linear | -0.0531 | 0.0407 | -0.1329 | 0.0266 | -1.3057 | 0.1917 |
|  | quadratic | 0.0022 | 0.0014 | -0.0004 | 0.0049 | 1.6334 | 0.1024 |
| GR | linear | 0.0148 | 0.0053 | 0.0043 | 0.0252 | 2.7584 | 0.0058 |
|  | quadratic | -0.0003 | 0.0002 | -0.0006 | 0.0000 | -1.7241 | 0.0847 |
| SOD | linear | -0.0408 | 0.0275 | -0.0946 | 0.0131 | -1.4837 | 0.1379 |
|  | quadratic | 0.0007 | 0.0006 | -0.0005 | 0.0020 | 1.1938 | 0.2326 |
| CAT | linear | -0.0566 | 0.0331 | -0.1215 | 0.0084 | -1.7070 | 0.0878 |
|  | quadratic | 0.0012 | 0.0007 | -0.0003 | 0.0026 | 1.6134 | 0.1067 |
| GST | linear | -0.0959 | 0.0945 | -0.2811 | 0.0893 | -1.0150 | 0.3101 |
|  | quadratic | 0.0031 | 0.0040 | -0.0046 | 0.0109 | 0.7894 | 0.4299 |
| GSH | linear | -0.0157 | 0.0099 | -0.0351 | 0.0037 | -1.5825 | 0.1135 |
|  | quadratic | 0.0004 | 0.0002 | 0.0000 | 0.0007 | 2.1015 | 0.0356 |
| MDA | linear | 0.0014 | 0.0039 | -0.0061 | 0.0090 | 0.3662 | 0.7142 |
|  | quadratic | -0.0001 | 0.0001 | -0.0002 | 0.0001 | -0.8599 | 0.3898 |
| 8-OHdG | linear | 0.0160 | 0.0083 | -0.0002 | 0.0322 | 1.9379 | 0.0526 |
|  | quadratic | -0.0003 | 0.0001 | -0.0006 | 0.0000 | -2.1281 | 0.0333 |
| PC | linear | 0.0056 | 0.0025 | 0.0007 | 0.0104 | 2.2490 | 0.0245 |
|  | quadratic | 0.0000 | 0.0001 | -0.0001 | 0.0001 | -0.4535 | 0.6502 |

**Supplementary Table S12.** **Regression table of neonicotinoids (neonics) effects at different concentrations.** Italic oxidative stress markers represent mRNA expression level of the respective enzyme. CI: 95% confidence interval; ROS: reactive oxygen species concentration; ^•^OH: hydroxyl concentration; CAT: catalase activity; SOD: superoxide dismutase activity; GST: glutathione-S-transferase activity; POD: peroxidase activity; CarE: carboxylesterase activity; GR: glutathione reductase activity; GSH: glutathione concentration; MDA: malondialdehyde concentration; 8-OHdG: [8-hydroxy-2-deoxyguanosine](https://www.ncbi.nlm.nih.gov/pmc/articles/PMC3261525/) concentration; PC: protein carbonyl group concentration.

| oxidative stress marker | term | estimate | standard error | CI lower bound | CI upper bound | Z value | P value |
| --- | --- | --- | --- | --- | --- | --- | --- |
| ROS | linear | -0.0105 | 0.0508 | -0.1101 | 0.0891 | -0.2071 | 0.8359 |
|  | quadratic | -0.0020 | 0.0027 | -0.0073 | 0.0033 | -0.7467 | 0.4552 |
| ^•^OH | linear | 0.1629 | 1.2318 | -2.2514 | 2.5772 | 0.1322 | 0.8948 |
|  | quadratic | 0.0030 | 0.0591 | -0.1128 | 0.1187 | 0.0501 | 0.9601 |
| CAT | linear | 0.1512 | 0.0547 | 0.0441 | 0.2584 | 2.7664 | 0.0057 |
|  | quadratic | 0.0065 | 0.0028 | 0.0010 | 0.0120 | 2.3216 | 0.0203 |
| SOD | linear | 0.2203 | 0.0854 | 0.0529 | 0.3876 | 2.5795 | 0.0099 |
|  | quadratic | 0.0096 | 0.0047 | 0.0004 | 0.0188 | 2.0432 | 0.0410 |
| GST | linear | 0.2901 | 0.0688 | 0.1552 | 0.4250 | 4.2146 | <.0001 |
|  | quadratic | 0.0147 | 0.0034 | 0.0080 | 0.0213 | 4.3324 | <.0001 |
| POD | linear | 0.1408 | 0.1308 | -0.1156 | 0.3972 | 1.0760 | 0.2819 |
|  | quadratic | 0.0046 | 0.0075 | -0.0102 | 0.0194 | 0.6114 | 0.5409 |
| CarE | linear | 0.0548 | 0.2128 | -0.3624 | 0.4719 | 0.2574 | 0.7968 |
|  | quadratic | 0.0028 | 0.0108 | -0.0183 | 0.0240 | 0.2634 | 0.7922 |
| GR | linear | -0.2278 | 0.1916 | -0.6032 | 0.1477 | -1.1890 | 0.2345 |
|  | quadratic | -0.0123 | 0.0097 | -0.0313 | 0.0067 | -1.2711 | 0.2037 |
| SOD | linear | 3.3389 | 3.1708 | -2.8757 | 9.5535 | 1.0530 | 0.2923 |
|  | quadratic | 0.1463 | 0.1572 | -0.1618 | 0.4545 | 0.9308 | 0.3520 |
| CAT | linear | -3.3282 | 3.1734 | -9.5479 | 2.8915 | -1.0488 | 0.2943 |
|  | quadratic | -0.1601 | 0.1572 | -0.4682 | 0.1479 | -1.0188 | 0.3083 |
| GST | linear | -4.8354 | 2.6978 | -10.1229 | 0.4521 | -1.7924 | 0.0731 |
|  | quadratic | -0.2035 | 0.1113 | -0.4217 | 0.0147 | -1.8283 | 0.0675 |
| GSH | linear | -0.1522 | 0.0729 | -0.2952 | -0.0092 | -2.0864 | 0.0369 |
|  | quadratic | -0.0064 | 0.0031 | -0.0125 | -0.0002 | -2.0274 | 0.0426 |
| MDA | linear | 0.2290 | 0.0781 | 0.0758 | 0.3821 | 2.9304 | 0.0034 |
|  | quadratic | 0.0093 | 0.0042 | 0.0011 | 0.0175 | 2.2239 | 0.0262 |
| 8-OHdG | linear | 1.0297 | 1.0969 | -1.1202 | 3.1795 | 0.9387 | 0.3479 |
|  | quadratic | 0.0425 | 0.0526 | -0.0606 | 0.1456 | 0.8078 | 0.4192 |
| PC | linear | 0.3638 | 0.3077 | -0.2392 | 0.9669 | 1.1824 | 0.2370 |
|  | quadratic | 0.0160 | 0.0150 | -0.0134 | 0.0455 | 1.0683 | 0.2854 |

**Supplementary Table S13. Regression table of clothianidin and imidacloprid on oxidative stress markers.** Clothianidin (262 data points) and Imidacloprid (249 data points) are the two most used neonicotinoids (neonics) in our data set. Italic oxidative stress markers represent mRNA expression level of the respective enzyme. CI: 95% confidence interval; ROS: reactive oxygen species concentration; ^•^OH: hydroxyl concentration; CAT: catalase activity; SOD: superoxide dismutase activity; GST: glutathione-S-transferase activity; POD: peroxidase activity; CarE: carboxylesterase activity; GR: glutathione reductase activity; GSH: glutathione concentration; MDA: malondialdehyde concentration; 8-OHdG: [8-hydroxy-2-deoxyguanosine](https://www.ncbi.nlm.nih.gov/pmc/articles/PMC3261525/) concentration; PC: protein carbonyl group concentration.

| neonics | oxidative stress markers | estimate | standard error | CI lower bound | CI upper bound | Z value | *P* value |
| --- | --- | --- | --- | --- | --- | --- | --- |
| clothianidin | ROS | 0.3881 | 0.1656 | 0.0635 | 0.7128 | 2.3431 | 0.0191 |
|  | CAT | 0.4352 | 0.1501 | 0.1410 | 0.7294 | 2.8994 | 0.0037 |
|  | SOD | 0.4046 | 0.1499 | 0.1107 | 0.6984 | 2.6981 | 0.0070 |
|  | GST | 0.1745 | 0.1499 | -0.1194 | 0.4685 | 1.1637 | 0.2445 |
|  | POD | 0.2653 | 0.1503 | -0.0293 | 0.5598 | 1.7652 | 0.0775 |
|  | MDA | 0.4103 | 0.1539 | 0.1088 | 0.7119 | 2.6669 | 0.0077 |
|  | PC | 0.3588 | 0.1657 | 0.0342 | 0.6835 | 2.1662 | 0.0303 |
| imidacloprid | ROS | 0.1438 | 0.0627 | 0.0209 | 0.2668 | 2.2926 | 0.0219 |
|  | CAT | -0.1778 | 0.0569 | -0.2894 | -0.0662 | -3.1233 | 0.0018 |
|  | SOD | 0.0494 | 0.0544 | -0.0571 | 0.1560 | 0.9092 | 0.3632 |
|  | GST | 0.1224 | 0.0654 | -0.0057 | 0.2505 | 1.8726 | 0.0611 |
|  | POD | -0.0654 | 0.0773 | -0.2168 | 0.0861 | -0.8459 | 0.3976 |
|  | CarE | -0.3163 | 0.0868 | -0.4864 | -0.1462 | -3.6440 | 0.0003 |
|  | *SOD* | -0.0663 | 0.1561 | -0.3723 | 0.2398 | -0.4243 | 0.6713 |
|  | *CAT* | 0.0585 | 0.1562 | -0.2476 | 0.3646 | 0.3749 | 0.7078 |
|  | *GST* | 0.0024 | 0.1089 | -0.2111 | 0.2158 | 0.0216 | 0.9828 |
|  | MDA | 0.0551 | 0.0631 | -0.0686 | 0.1787 | 0.8727 | 0.3828 |

**Supplementary Fig. S1** **In neonicotinoid contact tests only reactive oxygen species (ROS) and malondialdehyde (MDA) concentration were significantly increased.** Overall effects of neonicotinoids on the generation of ROS, antioxidant enzyme activities and expression, and oxidative stress-associated damage products determined via contact tests. k is equal to the number of data points and the number in brackets represents the number of studies. The black circle displays the mean, thicker black lines show 95% confidence intervals, and narrow lines show prediction intervals. Point sizes correlate with the inverse standard errors. Oxidative stress markers in bold significantly differ from 0; **P < 0.01. ROS: reactive oxygen species concentration; CAT: catalase activity; GST: glutathione-S-transferase activity; GSH: glutathione concentration; GR: glutathione reductase activity; MDA: malondialdehyde concentration.

**Supplementary Fig. S2 A PRISMA (Preferred Reporting Items for Systematic reviews and Meta-analyses) Flow diagram of the article selection process.** No raw data available refers to studies where no raw data was reported, the authors did not respond to the data request, and it was impossible to obtain raw data via the *metaDigitise* (1) package. NMP: Nano- and microplastic particles; neonic: neonicotinoid insecticides; TWPs: tire wear particles; ROS: Reactive Oxygen Species.

**Supplementary Fig. S3 Nano- and microplastic particles (NMP) induce the formation of reactive oxygen species (ROS) and alter oxidative stress responses on average including** **20 values a with sampling variance of 10^-7^.** Overall effects of NMP on the generation of ROS, antioxidant enzyme activities and expression, and oxidative stress-associated damage products. K: number of data points (number of studies in brackets). The black circle displays the mean, thicker black lines show 95% confidence intervals, and narrow lines show prediction intervals. These estimates were calculated using mixed meta regression models without intercept for each pollutant separately. Point sizes correlate with inverse standard errors. Oxidative stress markers in bold significantly differ from zero (no effect); *P < 0.05, **P < 0.01, ***P < 0.001. Italic oxidative stress markers represent mRNA expression levels of respective enzymes. ROS: reactive oxygen species concentration; CAT: catalase activity; SOD: superoxide dismutase activity; GST: glutathione-S-transferase activity; POD: peroxidase activity; TAC: total antioxidant capacity; GSH: glutathione concentration; MDA: malondialdehyde concentration; 8-OHdG: [8-hydroxy-2-deoxyguanosine](https://www.ncbi.nlm.nih.gov/pmc/articles/PMC3261525/) concentration.

**Supplementary Fig. S4 Statistical power of** **oxidative stress markers after nano- and microplastic particles (NMP) exposure assuming that NMP effects are independent of their properties and experimental designs.** Relationship between statistical power (percent of cases where a t-test will be significant) and sample size used for this t-test for each NMP oxidative stress marker. The means and standard deviations of all NMP oxidative stress markers were used for the t-tests. Red lines indicate the lowest sample size where the statistical power is equal to or greater than 0.8. ROS: reactive oxygen species concentration; CAT: catalase activity; SOD: superoxide dismutase activity; GST: glutathione-S-transferase activity; POD: peroxidase activity; TAC: total antioxidant capacity; GSH: glutathione concentration; MDA: malondialdehyde concentration; 8-OHdG: [8-hydroxy-2-deoxyguanosine](https://www.ncbi.nlm.nih.gov/pmc/articles/PMC3261525/) concentration. Individual plots were arranged using the ggpubr package (version 0.6.2; (2)).

**Supplementary Fig. S5 Statistical power of oxidative stress markers after neonicotinoid (neonic) exposure assuming that neonic effects are independent of their properties and experimental designs.** Relationship between statistical power (percent of cases where a t-test will be significant) and sample size used for this t-test for each neonic oxidative stress marker. The means and standard deviations of all neonic oxidative stress markers were used for the t-tests. Red lines indicate the lowest sample size where the statistical power is equal to or greater than 0.8. ROS: reactive oxygen species concentration; ^•^OH: hydroxyl concentration; CAT: catalase activity; SOD: superoxide dismutase activity; GST: glutathione-S-transferase activity; POD: peroxidase activity; CarE: carboxylesterase activity; GR: glutathione reductase activity; GSH: glutathione concentration; MDA: malondialdehyde concentration; 8-OHdG: [8-hydroxy-2-deoxyguanosine](https://www.ncbi.nlm.nih.gov/pmc/articles/PMC3261525/) concentration; PC: protein carbonyl group concentration. Individual plots were arranged using the ggpubr package (version 0.6.2; (2)).

**Supplementary Fig. S6 Effect of exposure time on oxidative stress measurements in experiments with nano- and microplastic (NMP).** Bubble plots illustrating the relationship between the log transformed ratio of means (logROM) and the exposure times in days. The regression lines show the mean logROM with 95% confidence intervals. Point sizes correlate with the inverse standard errors. Oxidative stress markers in bold significantly differ from 0 (P < 0.05) in their linear or quadratic term. Italic oxidative stress markers represent mRNA expression level of the respective enzyme. ROS: reactive oxygen species concentration; CAT: catalase activity; SOD: superoxide dismutase activity; GST: glutathione-S-transferase activity; POD: peroxidase activity; TAC: total antioxidant capacity; GSH: glutathione concentration; MDA: malondialdehyde concentration; 8-OHdG: [8-hydroxy-2-deoxyguanosine](https://www.ncbi.nlm.nih.gov/pmc/articles/PMC3261525/) concentration. The GSH concentration and GST mRNA expression level were modelled only with a linear term.

**Supplementary Fig. S7 Effects of nano- and microplastic (NMP) concentration on oxidative stress markers.** Bubble plots illustrating the relationship between the log transformed ratio of means (logROM) and the log transformed NMP concentrations in % w/w. The regression lines show the mean logROM with 95% confidence intervals. Point sizes correlate with the inverse standard errors. Oxidative stress markers in bold significantly differ from 0 (P < 0.05) in their linear or quadratic term. Italic oxidative stress markers represent mRNA expression level of the respective enzyme. ROS: reactive oxygen species concentration; CAT: catalase activity; SOD: superoxide dismutase activity; GST: glutathione-S-transferase activity; POD: peroxidase activity; TAC: total antioxidant capacity; GSH: glutathione concentration; MDA: malondialdehyde concentration; 8-OHdG: [8-hydroxy-2-deoxyguanosine](https://www.ncbi.nlm.nih.gov/pmc/articles/PMC3261525/) concentration.

**Supplementary Fig. S8 Nano- and microplastic particles (NMP) size is mostly unrelated to the effect of NMP on oxidative stress markers.** Bubble plots illustrating the relationship between the log transformed ratio of means (logROM) and the log transformed NMP sizes in mm. The regression lines show the mean logROM with 95% confidence intervals. Point sizes correlate with the inverse standard errors. Italic oxidative stress markers represent mRNA expression level of the respective enzyme. ROS: reactive oxygen species concentration; CAT: catalase activity; SOD: superoxide dismutase activity; GST: glutathione-S-transferase activity; POD: peroxidase activity; GSH: glutathione concentration; MDA: malondialdehyde concentration; 8-OHdG: [8-hydroxy-2-deoxyguanosine](https://www.ncbi.nlm.nih.gov/pmc/articles/PMC3261525/) concentration.

**Supplementary Fig. S9 Different nano- and microplastic particles (NMP) polymers have different effects on oxidative stress markers.** Effects of polystyrene (**A**) and low-density polyethylene (**B**) NMP on the generation of ROS, antioxidant enzyme activities and expression, and oxidative stress-associated damage products. k is equal to the number of data points and the number in brackets represents the number of studies. The black circle displays the mean, thicker black lines show 95% confidence intervals, and narrow lines show prediction intervals. Point sizes correlate with the inverse standard errors. Oxidative stress markers in bold significantly differ from 0; *P < 0.05, **P < 0.01, ***P < 0.001. Italic oxidative stress markers represent mRNA expression levels of respective enzymes. ROS: reactive oxygen species concentration; CAT: catalase activity; SOD: superoxide dismutase activity; GST: glutathione-S-transferase activity; POD: peroxidase activity; TAC: total antioxidant capacity; GSH: glutathione concentration; MDA: malondialdehyde concentration; 8-OHdG: [8-hydroxy-2-deoxyguanosine](https://www.ncbi.nlm.nih.gov/pmc/articles/PMC3261525/) concentration.

**Supplementary Fig. S10 Different nano- and microplastic particles (NMP) shapes have different effects on oxidative stress markers.** Effects of fragments (**A**) and bead shaped (**B**) NMP on the generation of ROS, antioxidant enzyme activities and expression, and oxidative stress-associated damage products. k is equal to the number of data points and the number in brackets represents the number of studies. The black circle displays the mean, thicker black lines show 95% confidence intervals, and narrow lines show prediction intervals. Point sizes correlate with the inverse standard errors. Oxidative stress markers in bold significantly differ from 0; *P < 0.05, **P < 0.01, ***P < 0.001. Italic oxidative stress markers represent mRNA expression levels of respective enzymes. ROS: reactive oxygen species concentration; CAT: catalase activity; SOD: superoxide dismutase activity; GST: glutathione-S-transferase activity; POD: peroxidase activity; TAC: total antioxidant capacity; GSH: glutathione concentration; MDA: malondialdehyde concentration; 8-OHdG: [8-hydroxy-2-deoxyguanosine](https://www.ncbi.nlm.nih.gov/pmc/articles/PMC3261525/) concentration.

**Supplementary Fig. S11 Most oxidative stress markers do not change over time during neonicotinoids (neonics) exposure.** Bubble plots illustrating the relationship between the log transformed ratio of means (logROM) and the exposure times in days. The regression lines show the mean logROM with 95% confidence intervals. Point sizes correlate with the inverse standard errors. Oxidative stress markers in bold significantly differ from 0 (P < 0.05) in their linear or quadratic term. Italic oxidative stress markers represent mRNA expression level of the respective enzyme. ROS: reactive oxygen species concentration; ^•^OH: hydroxyl concentration; CAT: catalase activity; SOD: superoxide dismutase activity; GST: glutathione-S-transferase activity; POD: peroxidase activity; CarE: carboxylesterase activity; GR: glutathione reductase activity; GSH: glutathione concentration; MDA: malondialdehyde concentration; 8-OHdG: [8-hydroxy-2-deoxyguanosine](https://www.ncbi.nlm.nih.gov/pmc/articles/PMC3261525/) concentration; PC: protein carbonyl group concentration.

**Supplementary Fig. S12 Different neonicotinoid (neonic) concentrations alter some oxidative stress markers.** Bubble plots illustrating the relationship between the log transformed ratio of means (logROM) and the log transformed neonic concentrations in % w/w. The regression lines show the mean logROM with 95% confidence intervals. Point sizes correlate with the inverse standard errors. Oxidative stress markers in bold significantly differ from 0 (P < 0.05) in their linear or quadratic term. Italic oxidative stress markers represent mRNA expression level of the respective enzyme. ROS: reactive oxygen species concentration; ^•^OH: hydroxyl concentration; CAT: catalase activity; SOD: superoxide dismutase activity; GST: glutathione-S-transferase activity; POD: peroxidase activity; CarE: carboxylesterase activity; GR: glutathione reductase activity; GSH: glutathione concentration; MDA: malondialdehyde concentration; 8-OHdG: [8-hydroxy-2-deoxyguanosine](https://www.ncbi.nlm.nih.gov/pmc/articles/PMC3261525/) concentration; PC: protein carbonyl group concentration.

**Supplementary Fig. S13 Different neonicotinoids (neonics) have different effects on oxidative stress markers.** Effects of clothianidin (**A**) and imidacloprid (**B**) on the generation of ROS, antioxidant enzyme activities and expression, and oxidative stress-associated damage products. k is equal to the number of data points and the number in brackets represents the number of studies. The black circle displays the mean, thicker black lines show 95% confidence intervals, and narrow lines show prediction intervals. Point sizes correlate with the inverse standard errors. Oxidative stress markers in bold significantly differ from 0; *P < 0.05, **P < 0.01, ***P < 0.001. Italic oxidative stress markers represent mRNA expression levels of respective enzymes. ROS: reactive oxygen species concentration; CAT: catalase activity; SOD: superoxide dismutase activity; GST: glutathione-S-transferase activity; POD: peroxidase activity; CarE: carboxylesterase activity; MDA: malondialdehyde concentration; PC: protein carbonyl group concentration.

**Supplementary Fig. S14 Statistical power of oxidative stress markers after nano- and microplastic particles (NMP) exposure assuming that NMP effects can dependent of their properties and experimental designs.** Shown is the relationship between statistical power (percent of cases where a t-test will be significant) and sample size used for this t-test. The means and standard deviations of all NMP property and experimental design combinations were used for the t-tests and the statistical power was averaged for each oxidative stress marker. Red lines indicate the lowest sample size where the statistical power is equal to or greater than 0.8. ROS: reactive oxygen species concentration; CAT: catalase activity; SOD: superoxide dismutase activity; GST: glutathione-S-transferase activity; POD: peroxidase activity; TAC: total antioxidant capacity; GSH: glutathione concentration; MDA: malondialdehyde concentration; 8-OHdG: [8-hydroxy-2-deoxyguanosine](https://www.ncbi.nlm.nih.gov/pmc/articles/PMC3261525/) concentration. Individual plots were arranged using the ggpubr package (version 0.6.2; (2)).

**Supplementary Fig. S15 Statistical power of oxidative stress markers after neonicotinoid (neonic) exposure assuming that neonic effects can depend on their properties and experimental designs.** Shown is the relationship between statistical power (percent of cases where a t-test will be significant) and sample size used for this t-test. The means and standard deviations of all neonic property and experimental design combinations were used for the t-tests and the statistical power was averaged for each oxidative stress marker. Red lines indicate the lowest sample size where the statistical power is equal to or greater than 0.8. ROS: reactive oxygen species concentration; ^•^OH: hydroxyl concentration; CAT: catalase activity; SOD: superoxide dismutase activity; GST: glutathione-S-transferase activity; POD: peroxidase activity; CarE: carboxylesterase activity; GR: glutathione reductase activity; GSH: glutathione concentration; MDA: malondialdehyde concentration; 8-OHdG: [8-hydroxy-2-deoxyguanosine](https://www.ncbi.nlm.nih.gov/pmc/articles/PMC3261525/) concentration; PC: protein carbonyl group concentration. Individual plots were arranged using the ggpubr package (version 0.6.2; (2)).

**References**

(1) Pick J.L.; Nakagawa S.; Noble D.W.A. Reproducible, flexible and high‐throughput data extraction from primary literature: the metaDigitise r package. *Methods Ecol Evol.* **2018**, 10, 426–431. <https://doi.org/10.1111/2041-210X.13118>.
(2) Kassambara A. *Ggpubr: ‘ggplot2’ based publication ready plots*. 2023. R package version 0.6.2. <https://CRAN.R-project.org/package=ggpubr>. (accessed 2025-10-28).
